# IVCDB: a comprehensive database of iridoviruses for epidemiology, genetic evolution, and disease management

**DOI:** 10.1093/nar/gkaf838

**Published:** 2025-08-28

**Authors:** Mincong Liang, Weiqiang Pan, Zhengqi Feng, Yangchi Cui, Hualong Su, Changjun Guo, Jianguo He

**Affiliations:** State Key Laboratory for Biocontrol & Southern Marine Science and Engineering Guangdong Laboratory (Zhuhai), Guangdong Province Key Laboratory of Aquatic Economic Animals, School of Life Sciences, Sun Yat-sen University, Guangzhou, Guangdong 510275, China; State Key Laboratory for Biocontrol & Southern Marine Science and Engineering Guangdong Laboratory (Zhuhai), Guangdong Province Key Laboratory of Aquatic Economic Animals, School of Life Sciences, Sun Yat-sen University, Guangzhou, Guangdong 510275, China; State Key Laboratory for Biocontrol & Southern Marine Science and Engineering Guangdong Laboratory (Zhuhai), Guangdong Province Key Laboratory of Aquatic Economic Animals, School of Life Sciences, Sun Yat-sen University, Guangzhou, Guangdong 510275, China; State Key Laboratory for Biocontrol & Southern Marine Science and Engineering Guangdong Laboratory (Zhuhai), Guangdong Province Key Laboratory of Aquatic Economic Animals, School of Life Sciences, Sun Yat-sen University, Guangzhou, Guangdong 510275, China; Institute of Ecology, College of Urban and Environmental Sciences, Peking University, Beijing 100871, China; State Key Laboratory for Biocontrol & Southern Marine Science and Engineering Guangdong Laboratory (Zhuhai), Guangdong Province Key Laboratory of Aquatic Economic Animals, School of Life Sciences, Sun Yat-sen University, Guangzhou, Guangdong 510275, China; State Key Laboratory for Biocontrol & Southern Marine Science and Engineering Guangdong Laboratory (Zhuhai), Guangdong Province Key Laboratory of Aquatic Economic Animals, School of Life Sciences, Sun Yat-sen University, Guangzhou, Guangdong 510275, China

## Abstract

Iridoviruses, a globally distributed group of double-stranded DNA viruses, exhibit remarkable environmental adaptability and host-switching capabilities, infecting over 200 species of ectothermic vertebrates and invertebrates, including reptiles, amphibians, fish, crustaceans, and insects. These characteristics have led to substantial economic losses in global aquaculture and population declines in amphibian and invertebrate species. Despite accumulating genomic data, research progress remains constrained by three critical limitations: fragmented multidimensional data resources, ambiguous taxonomic levels, and insufficient spatiotemporal visualization tools. To address these challenges, we present the Iridovirus Comprehensive Database (IVCDB; https://www.iridovirus.com/), a comprehensive database consolidating multidimensional iridovirus-related data. IVCDB provides curated comprehensive information on 310 geographically distinct virus isolates complemented by an interactive geospatial visualization system and a nonredundant protein database containing 4378 viral proteins, including 162 core genes/proteins, derived from a standardized genome annotation pipeline. This database also contains phylogenetic relationships across various viral taxonomic levels within the family *Iridoviridae*anda viral genome collinearity analysis tool, facilitating taxonomic analysis and supporting novel species identification. Furthermore, IVCDB integrates an application-oriented knowledge base including host range data, experimentally validated vaccines, and field-deployable visual detection methods. IVCDB offers substantial support for disease control and host conservation while addressing systemic bottlenecks in viral cross-species transmission research.

## Graphical Abstract

**Graphical Abstract:**
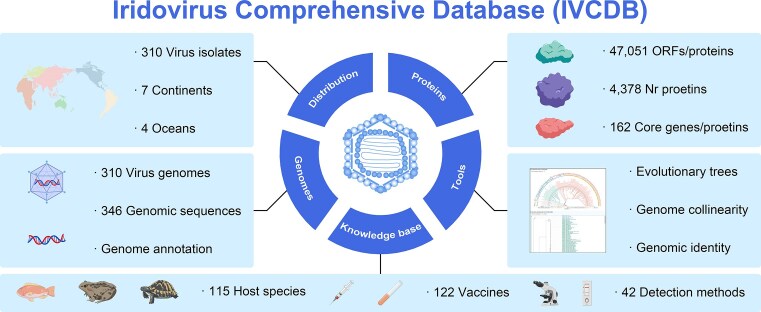

## Introduction

Iridoviruses, belonging to the family *Iridoviridae*, are large nucleocytoplasmic icosahedral viruses containing double-stranded DNA genomes [1, 2]. First documented in 1954 [3], these viruses exhibit a cosmopolitan distribution, with 310 isolates possessing complete or partial genomes having been reported across 34 countries and international waters on all seven continents as of May 2025. Recent investigations have revealed their ecological expansion into extreme environments, including Antarctic frigid waters [4] and deep-sea habitats [5, 6]. Advancements in molecular detection technologies [7, 8] have revealed the significant role of iridoviruses in dual crises affecting both global aquaculture [9, 10] and amphibian conservation [11]. Emerging evidence has also linked these viruses to population declines in various invertebrate species [12].

The application of advanced sequencing technologies, including metagenomics, has generated an expanding repository of iridovirus genomes [4, 5, 13]. However, critical gaps persist in integrating geospatial epidemiology data with molecular evolutionary patterns, significantly impeding research on cross-species transmission of iridoviruses and the development of disease control strategies. Although several iridovirus-related data resources exist, such as viral genomes and protein sequences in the NCBI RefSeq database [14] and the GenBank database [15], viral protein structures in the RCSB Protein Data Bank [16], viral taxonomic levels in the International Committee on Taxonomy of Viruses [1], and other databases containing limited content [17, 18], most databases narrowly focus on single-dimensional data and several key unresolved issues persist, including misclassified virus isolates, inadequate visualization of spatiotemporal distributions, and lack of standardized data description. These limitations stem from two systemic deficiencies: fragmented metadata architectures preventing interoperability between genomic and geospatial datasets, and the absence of specific analytical standards tailored for iridoviruses.

To address these critical gaps, we present the Iridovirus Comprehensive Database (IVCDB), a comprehensive database synthesizing multidimensional iridovirus-related data with significant advancements over conventional databases. IVCDB features four key innovations: (i) a curated dataset with detailed records for 310 virus isolates and an interactive geospatial visualization system for epidemiological tracking; (ii) a standardized genome annotation pipeline for iridoviruses and the implementation of open reading frame (ORF) reannotation for viral genomes, establishing a nonredundant protein database containing 4378 viral proteins including 162 core genes/proteins; (iii) maximum likelihood phylogenetic evolutionary relationships based on core genes/proteins combined with genome-wide nucleotide sequence identity analysis, enabling precise analysis of viral taxonomic levels and novel species discovery, supplemented by a viral genome collinearity analysis tool; and (iv) an application-oriented knowledge base encompassing host range data, experimentally validated vaccines, and field-deployable visual detection methods critical for epidemiological surveillance and aquaculture health management.

Building upon these resources, the IVCDB is positioned to become a central hub for accessing cutting-edge insights into iridoviruses and associated research domains. We anticipate that the database will facilitate advances across multiple biological dimensions in iridovirus research, ultimately enhancing disease control and wildlife conservation efforts. The IVCDB is freely accessible on the internet (https://www.iridovirus.com/) with planned annual updates.

## Materials and methods

### Data collection, verification, and management

Metadata for 310 iridovirus isolates with complete or partial genomes retrieved from the NCBI RefSeq database [14] and the GenBank database [15] prior to May 2025 were collected and verified. The metadata were standardized to include critical details for each virus isolate: name, host or sample source, collection time, geographic origin, and associated references. To address missing records in the GenBank database, mainstream search engines were used to identify references directly linked to virus isolates lacking associated references. Subsequently, metadata discrepancies or gaps were resolved by cross-checking details described in the original references. For metadata included in the original references, regardless of whether it was consistent with the records in the GenBank database, the original reference information took precedence. For virus isolates lacking the original references or where certain metadata were missing from the original references, the GenBank database records were retained. Meanwhile, the taxonomic levels of all virus isolates were recalibrated.

Geographic locations of virus isolates were formatted into a five-tier hierarchical format, excluding isolates with incomplete location data: specific site, city (county), province (state), country, and continent. Geographic coordinates (latitude and longitude) and elevation were appended to each entry (if available). Host or sample sources were annotated with conservation statuses retrieved from the International Union for Conservation of Nature Red List of Threatened Species (https://www.iucnredlist.org/) and NatureServe Explorer (https://explorer.natureserve.org/). For associated references, virus isolates were linked not only to publications describing genome sequencing and analyses but also to original reports documenting their initial discovery, collection, or isolation.

Moreover, associated references related to experimentally validated vaccines and field-deployable visual detection methods were collated through PubMed and Google Scholar.

### Interactive geospatial visualization system

Each virus isolate’s collection site was mapped onto a world map, featuring interactive filters and pop-up information panels. For virus isolates with precise latitude and longitude coordinates, their exact geographic coordinates were plotted directly on the map. For those lacking precise coordinates but having region-specific records, their locations represent the general regions where they were collected.

### Genome annotation, nonredundant protein extraction, core gene identification, and protein functional annotation

Drawing upon methods from predecessors’ studies [19–22], a standardized genome annotation pipeline for iridovirus genomes was established using the following criteria: (i) ORFs begin with ATG and end with TAA, TAG, TGA, TRA, or TAR, and contain at least 50 amino acids; (ii) both forward and reverse nested ORFs are excluded; and (iii) for overlapping co-directional ORFs, the longer ORF is retained, while overlapping ORFs of equal length are both preserved.

For nonredundant protein identification, all protein sequences translated from ORFs obtained through the annotation pipeline were extracted using CD-HIT v4.8.1 [23] with parameters “-c 0.5 -n 3” to construct a nonredundant protein database for the family *Iridoviridae*. Additionally, AlphaFold3 [24] was used to predict the structure of all nonredundant proteins.

Core gene/protein identification followed the protocols described by similar studies [25, 26]. The BLAST+ v2.16.0 [27] was used to compare the nonredundant proteins with those translated from ORFs in each virus isolate genome with parameters “-evalue 1e-10”. For *Iridoviridae*, *Alphairidovirinae*, and *Betairidovirinae* subgroups, proteins present in ≥75% of isolates were identified as core genes/proteins. For *Megalocytivirus* and *Ranavirus* subgroups, proteins showing 100% alignment across isolates were identified as core genes/proteins. When multiple nonredundant proteins aligned to identical viral proteins, those covering more isolates and having a lower average *E*-value were retained. For the other five genera (containing ≤10 virus isolates), core gene/protein identification was not performed due to the limited virus isolates and proteins.

Functional annotation of homologous superfamily, family, domain, and unintegrated family/domain was performed for all reannotated proteins from the standardized genome annotation pipeline and original proteins from the GenBank database for the iridovirus genomes, using InterProScan v5.75-106.0 [28] and databases including CATH-Gene3D v4.3.0, CDD v3.21, HAMAP v2025_01, NCBIFAM v17.0, PANTHER v19.0, Pfam v37.4, PIRSF v3.10, PRINTS v42.0, PROSITE patterns v2025_01, PROSITE profiles v2025_01, SMART v9.0, and SUPERFAMILY v1.75. Functional annotation of enzyme, orthology, and pathway was conducted using GhostKOALA v3.1 [29] with “Eukaryotes + Prokaryotes + Viruses” datasets.

### Phylogenetic evolutionary relationships analysis, genomic nucleotide sequence identity, and genome collinearity analysis

Following methodologies from previous studies [30, 31], phylogenetic evolutionary trees were constructed. For a given viral taxonomic level, BLAST+ was used to compare its core genes/proteins with the proteins translated from ORFs in each virus isolate within that level with parameters “-evalue 1e-10”. The BLAST results of each core gene/protein were consolidated, and multiple sequence alignment was performed using MAFFT v7.526 [32] with the L-INS-i algorithm. Subsequently, phylogenetic evolutionary trees were constructed using IQ-TREE v3.0.1 [33] with parameters “-m MFP+MERGE -B 1000”, incorporating partition models [34], model selection [35], and ultrafast bootstrap approximation [36]. The resulting trees were visualized using iTOL [37]. The subfamilies, genera, and species of all virus isolates were recalibrated based on the phylogenetic evolutionary trees and were double-checked using VISTA [38]. Genotyping of infectious spleen and kidney necrosis virus, Ambystoma tigrinum virus, frog virus 3, epizootic haematopoietic necrosis virus, and common midwife toad virus was conducted following the classification framework for the genus *Megalocytivirus* [30, 39] and the genus *Ranavirus* [31].

Genomic nucleotide sequence identity analysis involved 307 complete iridovirus genomes. To eliminate artifacts caused by terminal redundancies [1] and potential reverse complementation, all genomic sequences were standardized by adjusting reverse-complemented sequences and repositioning the terminal repeats, ensuring the major capsid protein gene was the first genomic feature (from 5′ to 3′). Pairwise global alignment of standardized genomes was then performed using the PairwiseAligner in Biopython v1.85 [40] to calculate the nucleotide sequence identity.

Genomic collinearity analysis was conducted using MUMmer v4.0.1 [41], where collinear regions between selected viral genomic sequences were identified through nucmer with the parameter “-l 10”, followed by visualization using mummerplot and gnuplot v5.4.2.

### Detailed pages for virus isolates and proteins

Detailed pages for each virus isolate containing standardized critical metadata, related pictures, and the annotation results through the standardized genome annotation pipeline were established. The reannotated proteins from the standardized genome annotation pipeline and the original proteins from the GenBank database for the virus isolate are presented in tabular form. Among them, the genomic annotation results were visualized using IGV [42]. Meanwhile, detailed pages containing sequence information and gene context for each reannotated protein and original protein from the GenBank database were established, including displaying the protein structure, functional annotation, and localization in the genome by PDBe Molstar [43], Feature-Viewer [44], and IGV.

## Results

### Database overview

The Iridovirus Comprehensive Database (IVCDB) comprises five main components: an interactive geospatial visualization system for iridovirus isolates, an iridovirus dataset containing detailed records and reannotated genomes per isolate, a nonredundant protein database with core genes/proteins, evolutionary relationship analysis tools for iridoviruses, and an application-oriented knowledge base containing host range data, experimentally validated vaccines, and field-deployable visual detection methods. In the current release, IVCDB systematically integrates standardized information from 310 iridovirus isolates with well-characterized genome sequences collected from all seven continents. Building upon these curated datasets, IVCDB provides researchers with a highly intuitive web interface for browsing and retrieving cutting-edge information on iridovirus research.

### Viral distribution

**Figure 1.**
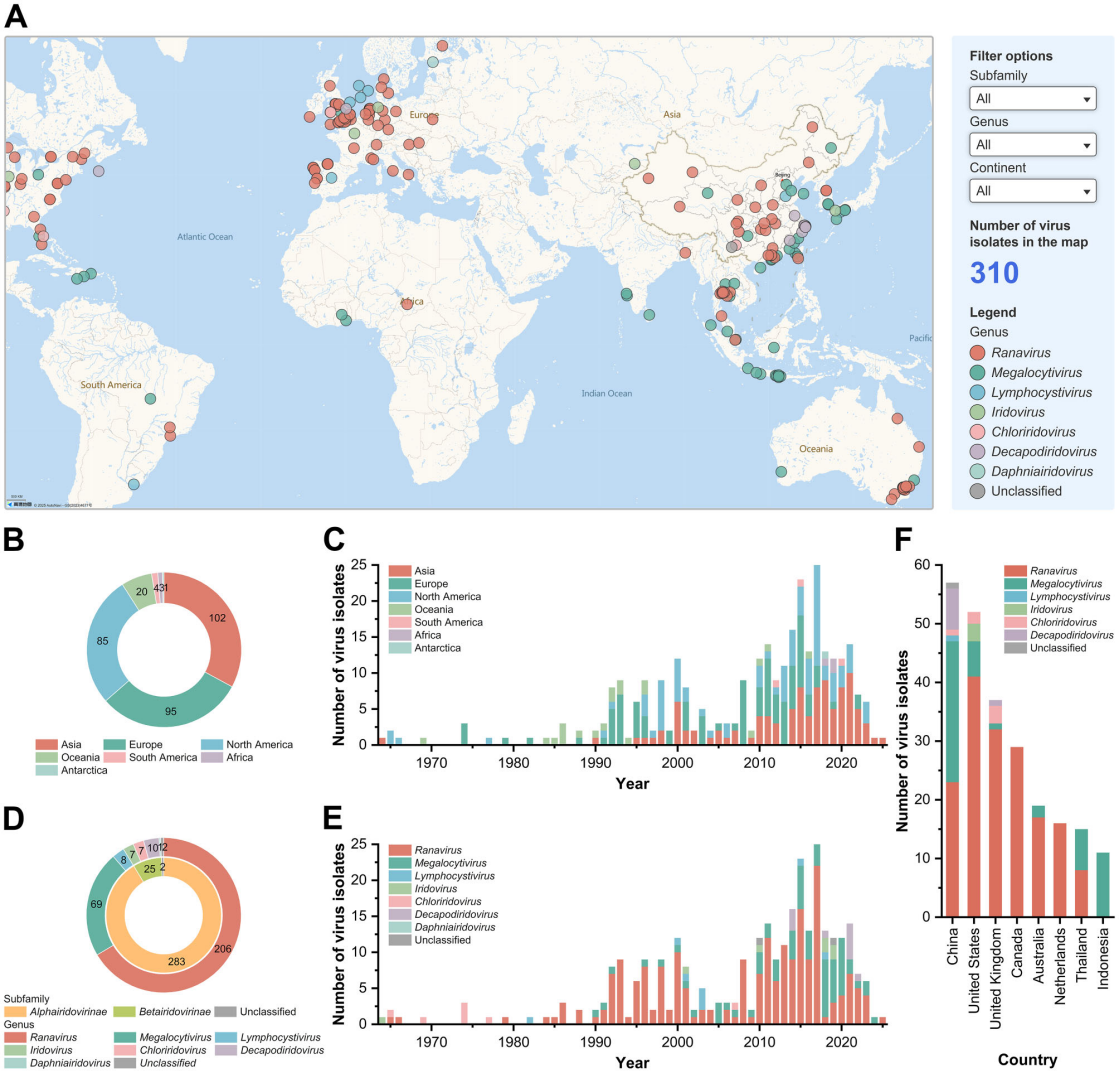
An interactive geospatial visualization system for virus isolates and statistical information of virus isolates. (**A**) Interface of the interactive geospatial visualization system for virus isolates. (**B**) Statistical distribution of virus isolates categorized by source. (**C**) Temporal distribution of virus isolate discoveries across continents. (**D**) Statistical classification of virus isolates based on taxonomic levels. (**E**) Temporal distribution of virus isolate discoveries by taxonomic level. (**F**) Classification information of virus isolates from countries contributing >10 virus isolates.

The interactive geospatial visualization system in IVCDB delineates the global distribution of iridovirus isolates, with color-coded differentiation across viral genera (Fig. 1A). Users can filter and check detailed metadata for each virus isolate, including taxonomic levels, host or sample source, collection time, and geographic coordinates.

Geospatial distribution analysis showed that most of the virus isolates were from Asia (32.9%), Europe (30.6%), and North America (27.4%) (Fig. 1B). Since 2000, Asia, Europe, and North America have been the primary sites of discovery for the vast majority of iridoviruses (Fig. 1C). This phenomenon may result from both increased viral dissemination due to expanding aquaculture and enhanced diagnostic capabilities. Taxonomic analysis identifies the subfamily *Alphairidovirinae* as the predominant subfamily (91.3%), particularly the genus *Ranavirus* (66.5%) and the genus *Megalocytivirus* (22.3%) (Fig. 1D). Although early research focused on the subfamily *Betairidovirinae* [3, 45], the genus *Ranavirus* emerged as the dominant genus following the first complete genome characterization [22]. Around 2010, the proportion of the genus *Megalocytivirus* began to increase after its initial genomic report [21], alongside discoveries of novel genera, including the genus *Decapodiridovirus* [46, 47] and the genus *Daphniairidovirus* [48], through advanced characterization (Fig. 1E). Notably, China, the United States, the United Kingdom, Canada, Australia, the Netherlands, Thailand, and Indonesia each contributed over 10 virus isolates. Country-specific patterns reflect different characteristics: the genus *Megalocytivirus*, which infects fish, accounts for >40% of virus isolates in major Asian aquaculture nations (China, Thailand, Indonesia) [30, 49], whereas the predominance of the genus *Ranavirus* in the United States, the United Kingdom, Canada, Australia, and the Netherlands correlates with extensive amphibian wild population surveillance [50–54] (Fig. 1F). Jamie Bojko [4] used metagenomic technology to identify a *Betairidovirinae* isolate from Antarctic deep-sea samples, suggesting the global distribution may be more extensive than previously estimated.

It should be noted that temporal and geographic distribution interpretations may be confounded by sequencing sample bias. The limited virus isolates from Africa and South America likely reflect limited research infrastructure and insufficient scientific attention rather than true epidemiological patterns.

### Genes and proteins

**Figure 2.**
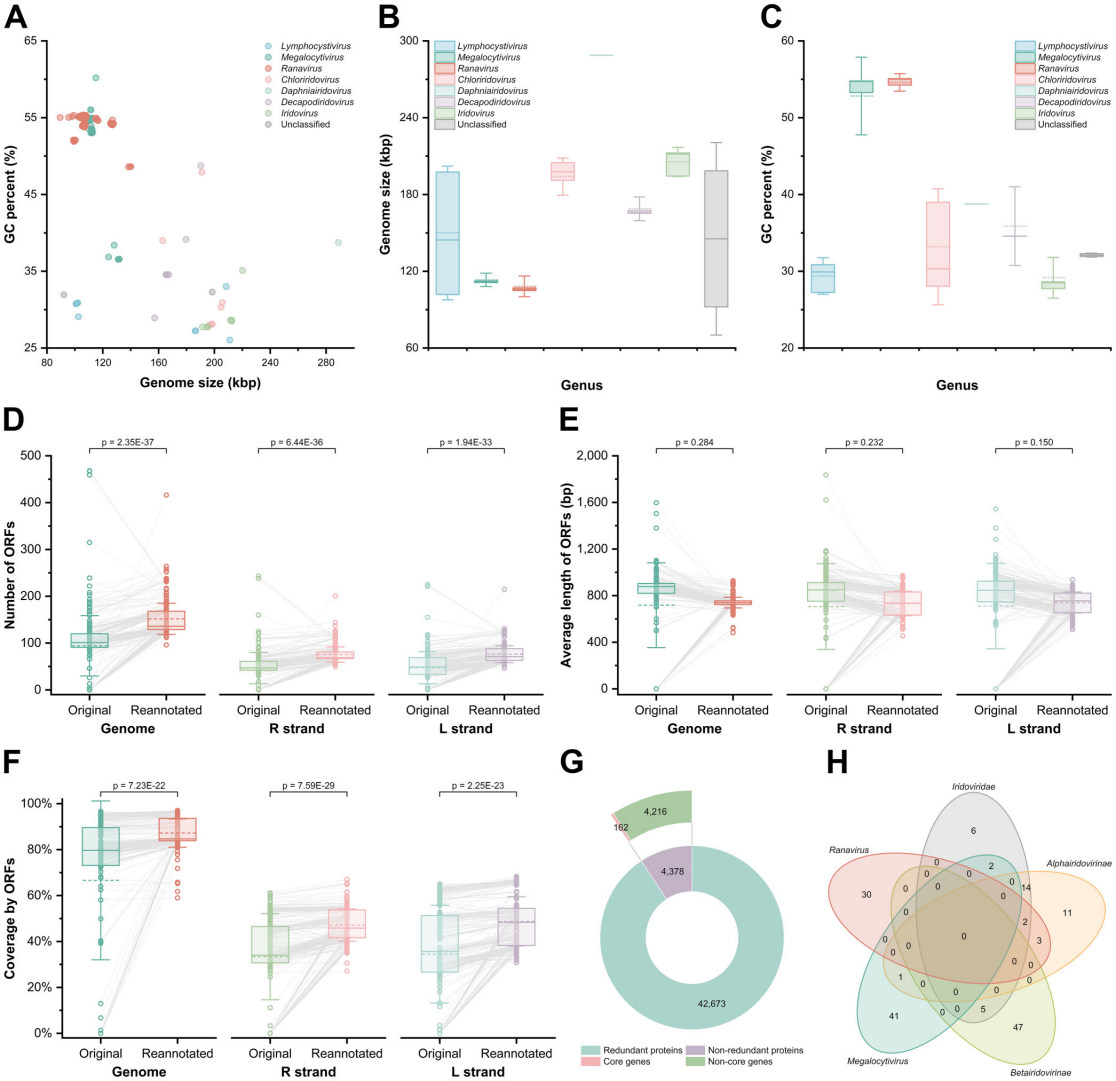
Statistical information of the standardized genome annotation pipeline and core genes. (**A**) Genome size and GC percent of iridoviruses. (**B**) Genome size of iridoviruses. (**C**) GC percent of iridoviruses. (**D**) Number of ORFs in virus isolates before and after reannotation. (**E**) Average length of ORFs in virus isolates before and after reannotation. (**F**) Genomic ORF coverage in virus isolates before and after reannotation. (**G**) Proportions of core genes/proteins and nonredundant proteins. (**H**) Intersection of core genes across viral taxonomic levels, demonstrating that a single core gene may belong to two or more viral taxonomic levels. In box plots, the box spans from the upper to the lower quartiles, whiskers represent standard deviations, the solid line inside the box indicates the median, and the dashed line denotes the mean value.

The statistical results of the viral genomes’ characteristics show that viruses of the genus *Ranavirus* and the genus *Megalocytivirus* have smaller genomic sizes and higher GC percentages (Fig. 2A). Viruses of the genus *Lymphocystivirus*, which belong to the subfamily *Alphairidovirinae*, have characteristics more similar to those of the subfamily *Betairidovirinae* (Fig. 2B and C). Although existing studies have annotated the genomes of some iridoviruses [19–22], the genomic annotations of many iridovirus isolates remain incomplete or entirely lacking. To enhance the standardization of genomic analysis for iridoviruses, a standardized genome annotation pipeline for iridovirus genomes has been developed, and ORFs for 310 virus isolates have been reannotated. Comparative analysis revealed that the average number of ORFs per virus isolate significantly increased from 94.2 to 151.8 after reannotation (Fig. 2D), while the average ORF length showed no significant change (Fig. 2E). Furthermore, ORF coverage across the genome significantly improved from 66.6% to 87.2% (Fig. 2F), a trend consistent across both the R and L strands. These findings underscore the necessity of a standardized genome annotation pipeline, which not only enhances ORF delineation for unannotated or partially annotated viral genomes but also facilitates the discovery of shorter or overlapping ORFs. Users can visualize original and reannotated ORFs via the IGV genome browser on each virus isolate’s detailed record page or download reannotated ORF sequences and translated protein sequences through the database’s download interface.

Following reannotation of virus isolates, 47 051 ORFs were identified. After translating these ORFs into proteins and merging sequences, a nonredundant iridovirus protein database comprising 4378 proteins was established (Fig. 2G). This database serves as a condensed yet representative resource for all iridovirus-encoded proteins, supporting comparative analyses of newly discovered isolates and metagenomic studies. Users can explore and search the nonredundant protein database through a dedicated data table (Supplementary Fig. S1) and can also view information, including protein features and gene context, on the detail page of each nonredundant protein. IVCDB also offers downloadable three-dimensional protein structures for each nonredundant protein to facilitate downstream analyses.

Furthermore, leveraging the nonredundant protein database, 162 core genes/proteins were identified across distinct viral taxonomic levels (Fig. 2G), including 29 core genes/proteins for the family *Iridoviridae*, 31 for the subfamily *Alphairidovirinae*, 52 for the subfamily *Betairidovirinae*, 44 for the genus *Megalocytivirus*, and 35 for the genus *Ranavirus*. The total number of core genes/proteins is less than the sum of the category-specific counts because individual genes/proteins may serve as core genes/proteins across multiple viral taxonomic levels (Fig. 2H). Redefining iridovirus core genes/proteins will advance our understanding of phylogenetic relationships among iridoviruses and provide a robust framework for characterizing unclassified iridoviruses. Users can access core gene/protein lists for specific taxonomic groups on dedicated pages (Supplementary Fig. S2).

### Detailed records for virus isolates and nonredundant proteins

**Figure 3.**
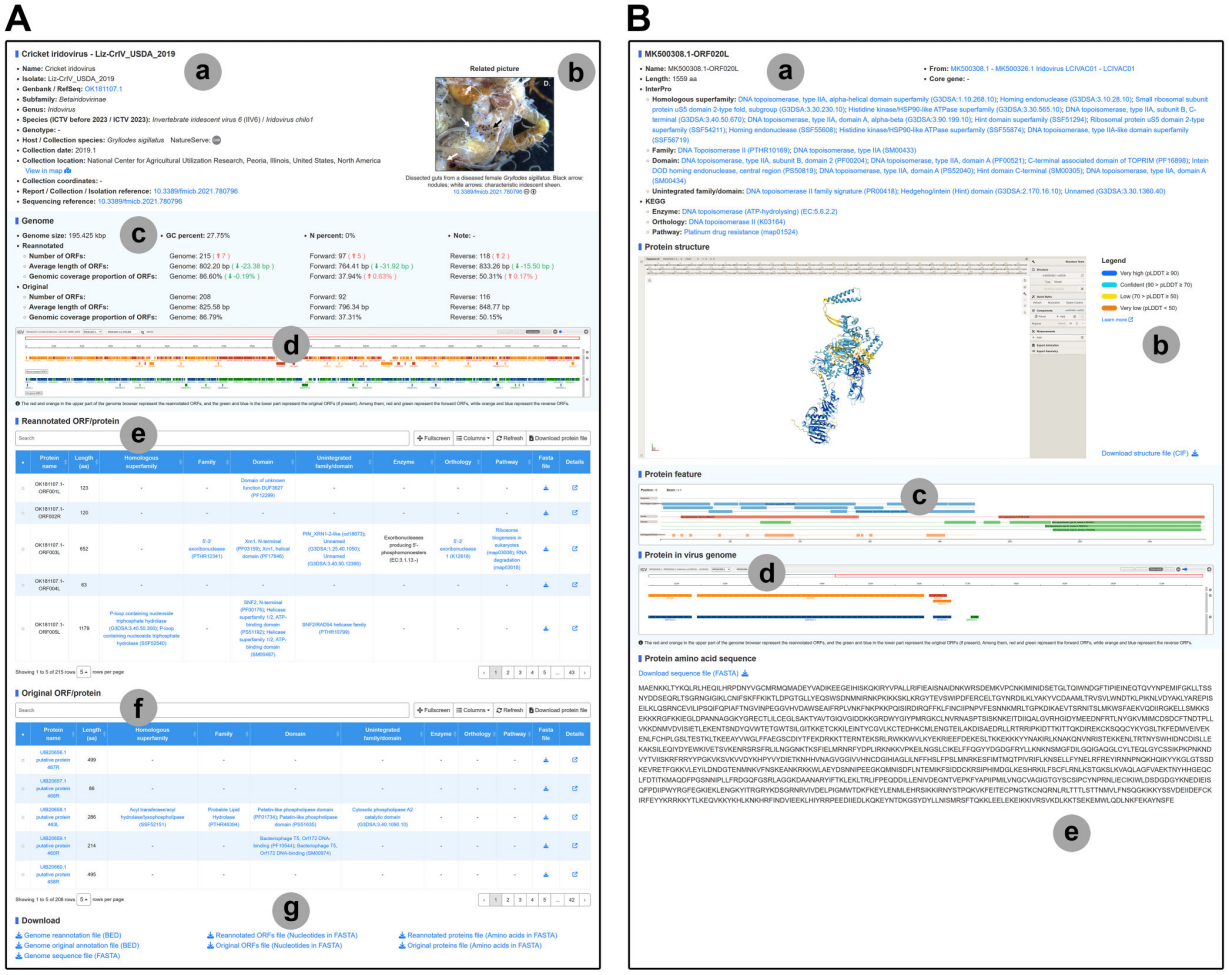
Detailed pages for each virus isolate and protein. (**A**) Detail page for virus isolate exemplified by “Cricket iridovirus - Liz-CrIV_USDA_2019 (OK181107.1)”. “a” represents the standardized virus isolate data; “b” represents the related picture; “c” represents the genome details of the virus isolate; “d” represents the IGV genome browser that shows results of reannotation alongside original annotations; “e” represents the reannotated ORFs/proteins from the standardized genome annotation pipeline; “f” represents the original ORFs/proteins from the GenBank database; and “g” represents the download link. (**B**) Detail page for protein exemplified by MK500308.1-ORF020L. “a” represents the standardized protein data; “b” represents the three-dimensional structure of the protein and the download link; “c” represents the protein features; “d” represents the gene context of the protein visualized in the IGV genome browser; and “e” represents the amino acid sequence of the protein and the download link.

IVCDB curates comprehensive records for each virus isolate, compiling critical metadata including summary information and genome details into an optimized tabular format for visual inspection and data retrieval (Supplementary Figs S3 and S4). Standardized data fields in detailed records are derived from original references or the GenBank database records. The summary information includes (Fig. 3A) (i) virus nomenclature and isolate designation, (ii) GenBank and RefSeq accession number, (iii) revised viral taxonomic levels, (iv) host species or sample source with degree of endangerment, (v) collection date, (vi) collection location in five-tier hierarchical format, (vii) precise geospatial coordinates (if available), (viii) original references for report/collection/isolation and genome sequencing (if available), and (ix) related images such as host tissue sections or electron micrographs of virions with notes from references. The genome details include (Fig. 3A) (i) genomic basic information including length and GC percentage, (ii) comparison of ORF annotation results from the standardized genomic annotation pipeline with those in the GenBank database, (iii) visualized new annotation results and the original annotation using IGV, and (iv) download links for the genome sequence file, genome annotation files, ORFs’ sequence files, and protein sequence files. Meanwhile, the reannotated proteins from the standardized genome annotation pipeline and the original proteins from the GenBank database for the virus isolate with the functional annotations are presented in tabular form.

IVCDB also establishes detailed records for 47 051 reannotated proteins and 29 208 original proteins from the GenBank database. Each record contains (Fig. 3B) (i) standardized protein data, (ii) three-dimensional structure of the protein (only for the nonredundant proteins in the current version), (iii) protein functional annotation from the InterProScan in a visual form, (iv) positions of the protein in the genome through the IGV genome browser, and (v) amino acid sequence of the protein and the download link for the sequence file.

### Viral phylogenetic evolutionary relationships

**Figure 4.**
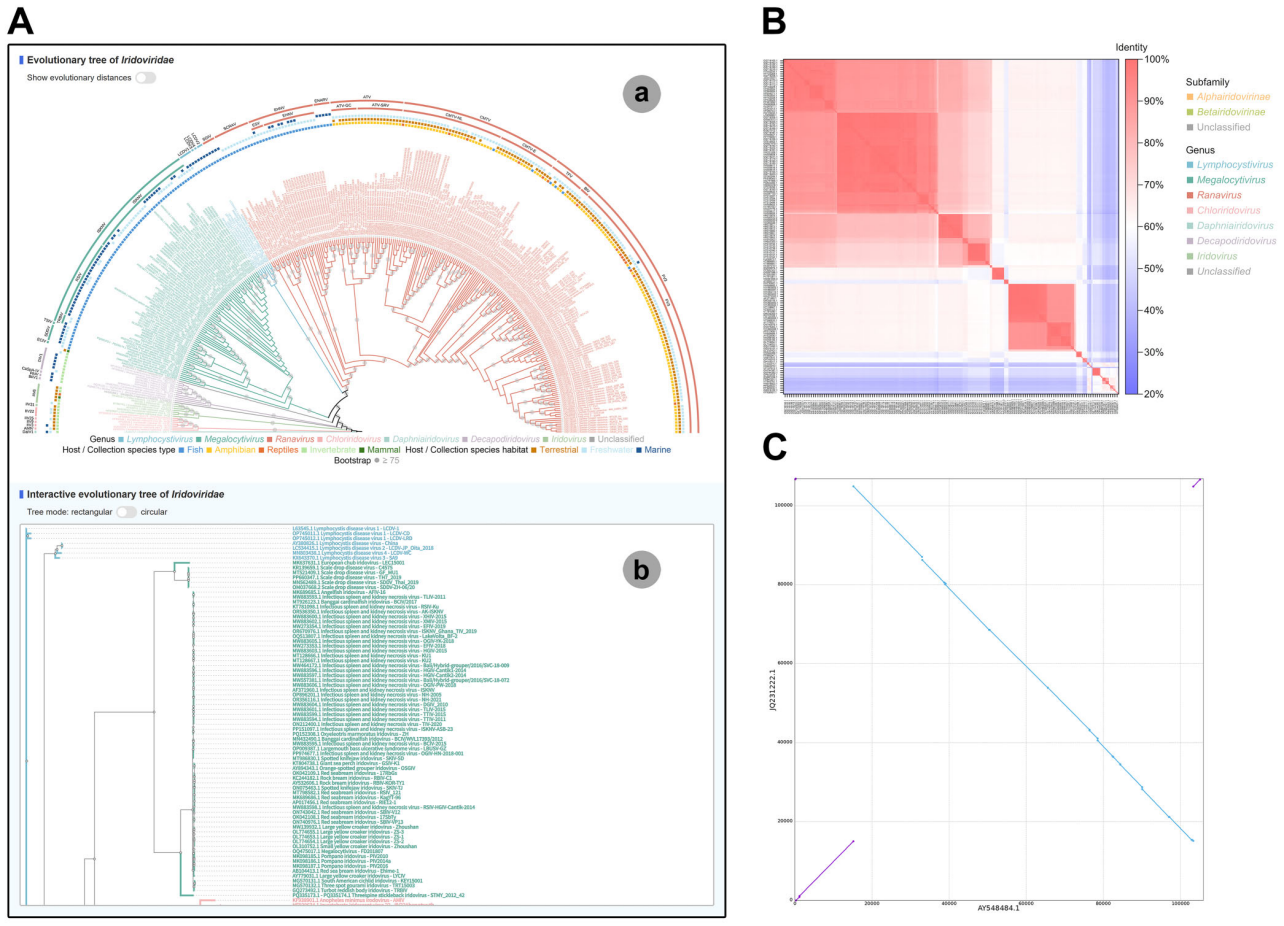
Phylogenetic and genomic analysis tools. (**A**) Evolutionary tree page exemplified by the family *Iridoviridae*. “a” represents the pre-constructed evolutionary tree, and “b” represents the interactive evolutionary tree. (**B**) Genomic pairwise identity calculated from corrected complete iridovirus genomes. (**C**) Genomic collinearity exemplified by “Frog virus 3 - FV3 (AY548484.1)” versus “Common midwife toad virus - Mesotriton alpestris/2008/E (JQ231222.1).” A line connecting two dots represents a syntenic region between the genomes. The purple lines indicate forward strand comparisons, and the blue lines indicate reverse strand comparisons.

Based on the 162 core genes across distinct viral taxonomic levels identified by IVCDB, phylogenetic evolutionary trees were reconstructed for the family *Iridoviridae*, the subfamily *Alphairidovirinae*, the subfamily *Betairidovirinae*, the genus *Megalocytivirus*, and the genus *Ranavirus* (Supplementary Figs S5–S9). These trees integrate host or sample source information for virus isolates, allowing users to view and interact with them (Fig. 4A). The phylogenetic evolutionary trees robustly reflect phylogenetic evolutionary relationships among iridovirus isolates within the family *Iridoviridae*. Moreover, IVCDB provides genome-wide nucleotide sequence identity derived from corrected genomes to aid viral classification (Fig. 4B).

IVCDB also incorporates a genome collinearity analysis system critical for studying viral genome evolution (Supplementary Fig. S10). Users can investigate collinearity relationships between any two genomic sequences of virus isolates (Fig. 4C). For iridoviruses, genomic rearrangements may play a significant role in their broad, variable host ranges and virulence shifts, particularly in ranaviruses.

Based on phylogenetic evolutionary trees, nucleotide sequence identity, and genome collinearity analyses, IVCDB supports the hypothesis that European chub iridovirus [55] and threespine stickleback iridovirus [56] represent novel species within the genus *Megalocytivirus*, while bivalve iridovirus 1 [57], carnivorous sponge-associated iridovirus [5], and Pentanymphon antarcticum iridovirus [4] likely represent novel species within the genus *Decapodiridovirus*. Furthermore, leveraging expanded core gene sets and refined taxonomic levels, the subfamilies, genera, and species of all virus isolates were recalibrated. This led to revisions in the taxonomic levels (subfamily and/or genus) for 12 virus isolates, including the four aforementioned novel species candidates (European chub iridovirus, bivalve iridovirus 1, carnivorous sponge-associated iridovirus, and Pentanymphon antarcticum iridovirus) (Supplementary Table S1). Species-level revisions were applied to 87 isolates, including the aforementioned threespine stickleback iridovirus as a potential novel species (Supplementary Table S2). Validation using VISTA also supported the revised virus taxonomic levels.

### Application-oriented knowledge base

Given the significant threat posed by iridoviruses to aquaculture and wildlife populations, professionals (including virologists) and nonspecialists (such as aquaculturists, conservationists, and science communicators) alike require access to the latest advancements in this field. To address this, IVCDB provides an intuitive knowledge base featuring host range data (Supplementary Fig. S11), experimentally validated vaccines (Supplementary Fig. S12), field-deployable visual detection methods (Supplementary Fig. S13), and links to original references for all data. Users can navigate content via a card-style, user-friendly interface and perform comparative analyses using standardized data tables.

In the current version, the knowledge base documents 115 host species (where a single species may be infected by multiple iridoviruses) across 49 fish, 36 amphibians, 13 reptiles, and 17 invertebrates. Notably, this includes four critically endangered, three endangered, seven vulnerable, and five near-threatened animal species. The knowledge base also includes 122 iridovirus vaccine studies involving different vaccine types and immunized animals, comprising 3 live vaccines, 5 attenuated vaccines, 43 inactivated vaccines, 43 subunit vaccines, 25 plasmid DNA vaccines, and 3 viral vectored vaccines, with 4 specifically designed for amphibians. While numerous laboratory detection methods exist for iridoviruses, field-deployable, low-cost, and visual rapid detection tools are critical for aquaculture and wildlife monitoring. The knowledge base integrates 42 advanced field-deployable visual detection methods targeting the genus *Lymphocystivirus*, the genus *Megalocytivirus*, the genus *Ranavirus*, and the genus *Decapodiridovirus*, offering practical solutions for disease control and wildlife protection.

## Discussion

As widespread pathogens, iridoviruses infect over 200 species of ectothermic vertebrates and invertebrates, including multiple threatened animal species [58]. Their prevalence poses significant threats to aquaculture [59], wildlife conservation [60], live animal trade, and import–export quarantine protocols [61], garnering increasing global attention. Iridoviruses also attract scientific interest due to their highly methylated, large double-stranded DNA genomes [62], which exhibit remarkable plasticity through genomic rearrangements and the presence of genomic repetitive sequences [63, 64]. Their ecological success stems from complex host-switching mechanisms operating across ectothermic vertebrates [26, 31, 65] and invertebrates [66, 67]. Thus, a comprehensive understanding of iridoviruses is critical for disease control, wildlife protection, and viral evolutionary studies.

IVCDB is the first multidimensional integrated database dedicated to iridoviruses, with a focus on spatiotemporal distribution, evolutionary relationships, and genomic insights. It offers a user-friendly platform with efficient data browsing, retrieval, and download functionalities, and provides standardized datasets that serve as a resource for iridovirus-related research. By implementing a standardized genome annotation pipeline, IVCDB delivers enhanced and detailed annotations for each virus isolate genome and refines core genes/proteins. Integrated phylogenetic evolutionary trees, nucleotide sequence identity, and genome collinearity analyses enable accurate viral classification and support the identification of potential novel species. Additionally, consolidated host range data, experimentally validated vaccines, and field-deployable visual detection methods provide valuable information for researchers, helping to guide research directions and focus research scopes.

In the future, IVCDB will be maintained and updated by incorporating additional iridovirus-related data, such as transcriptomic, metabolomic, and proteomic profiles. Publicly available high-throughput sequencing (HTS) datasets may contain numerous uncharacterized iridoviruses. Future efforts will leverage public HTS data to elucidate iridovirus epidemiology and evolution, offering valuable insights for further research. Concurrently, IVCDB will undergo annual updates to ensure data consistency and accuracy, and provide access to the latest information.

## Supplementary Material

gkaf838_Supplemental_File

## Data Availability

IVCDB is publicly accessible at https://www.iridovirus.com/, and all data are available online.
