# Supplementary material for: IVCDB: a comprehensive database of iridoviruses for epidemiology, genetic evolution, and disease management
Source: Nucleic Acids Res. 2025 Aug 28;54(D1):D790–800. doi: 10.1093/nar/gkaf838 (PMC12807783; doi:10.1093/nar/gkaf838)
Supplement: gkaf838_Supplemental_File [file gkaf838_supplemental_file.docx]

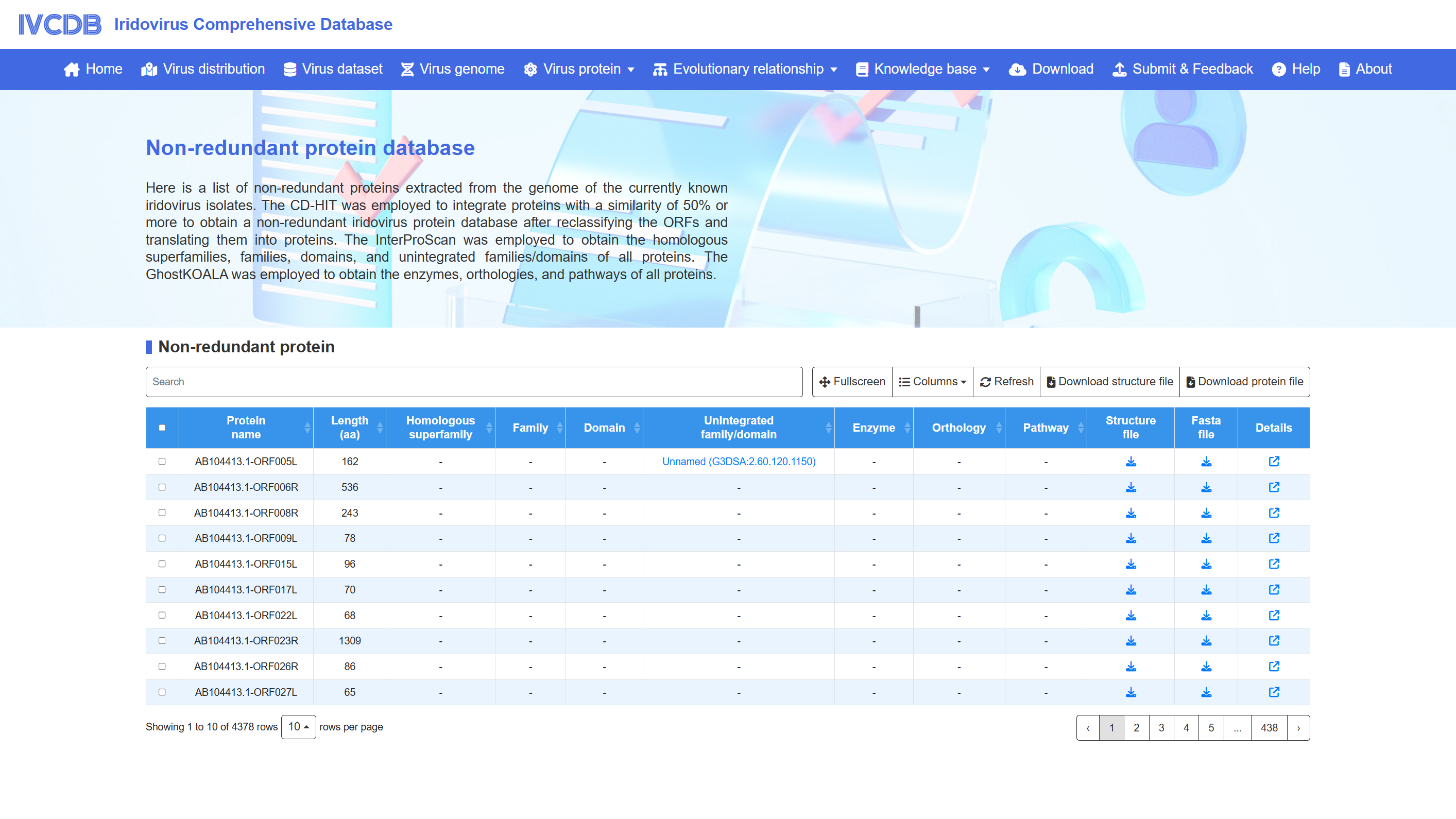


**Supplementary Figure S1.** Navigation interface in tabular form of the non-redundant protein database.


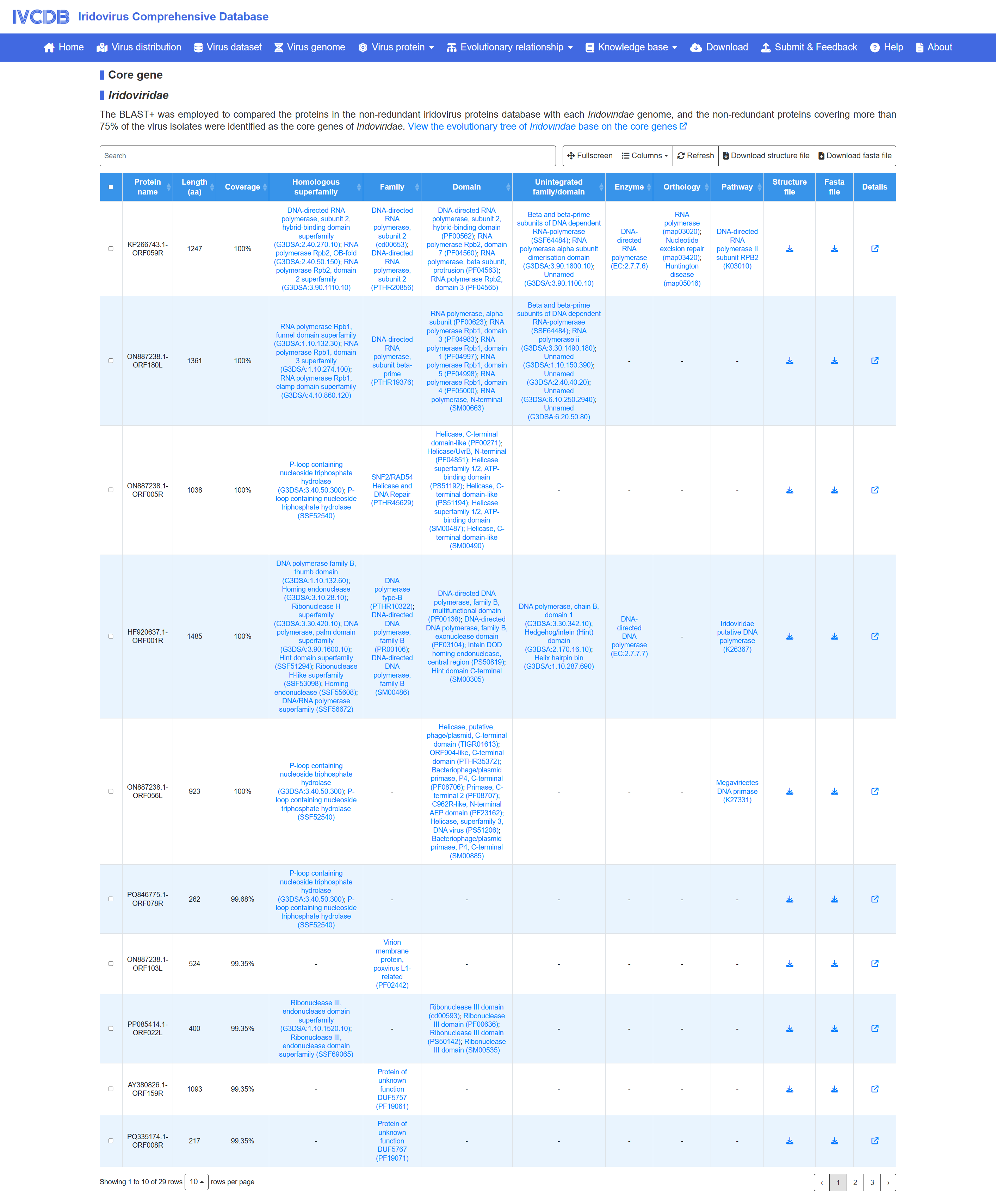


**Supplementary Figure S2.** Navigation interface in tabular form of the core genes/proteins (exemplified by the core genes/proteins of the family *Iridoviridae*).


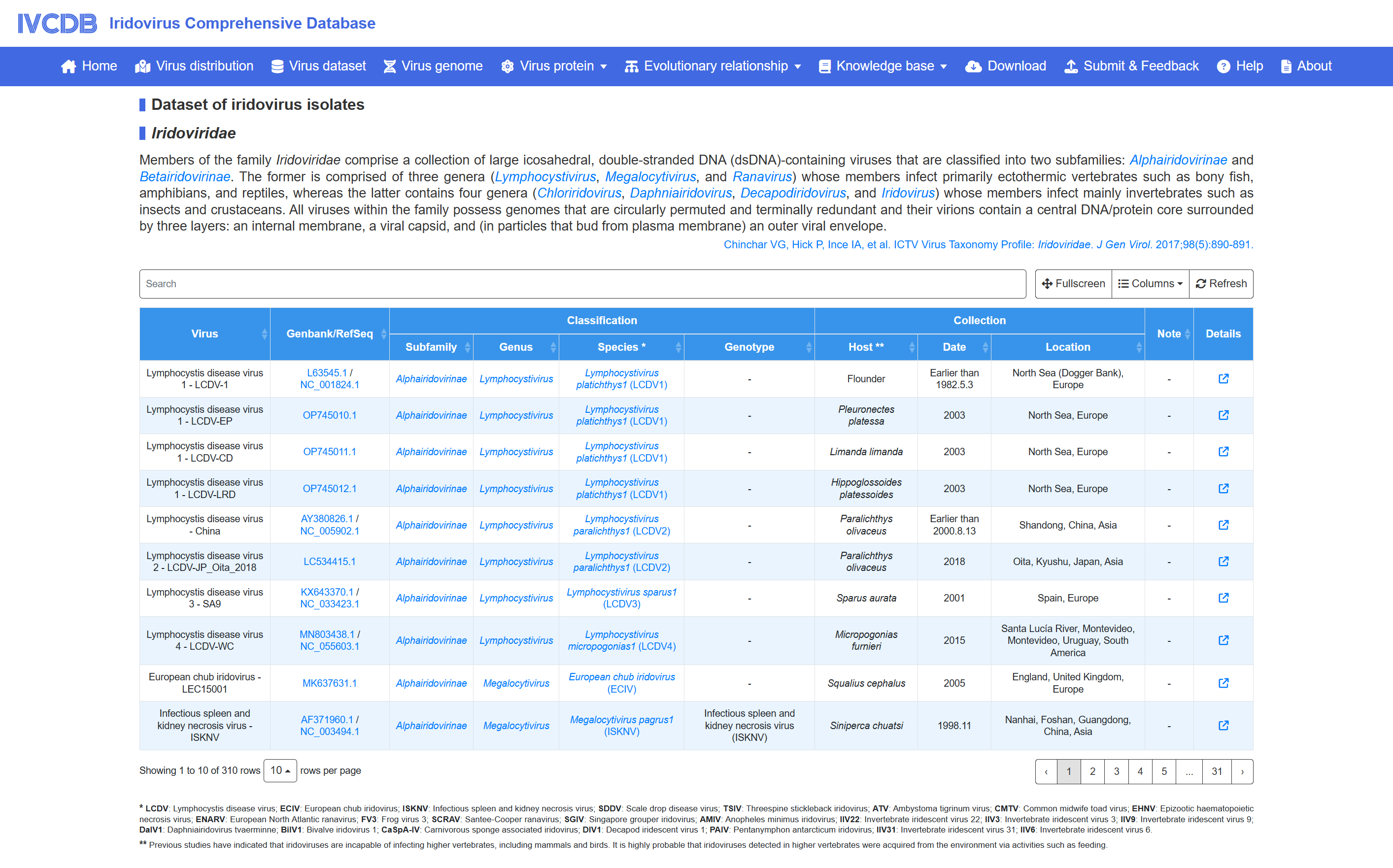


**Supplementary Figure S3.** Navigation interface in tabular form of the virus dataset (exemplified by the virus dataset of the family *Iridoviridae*).


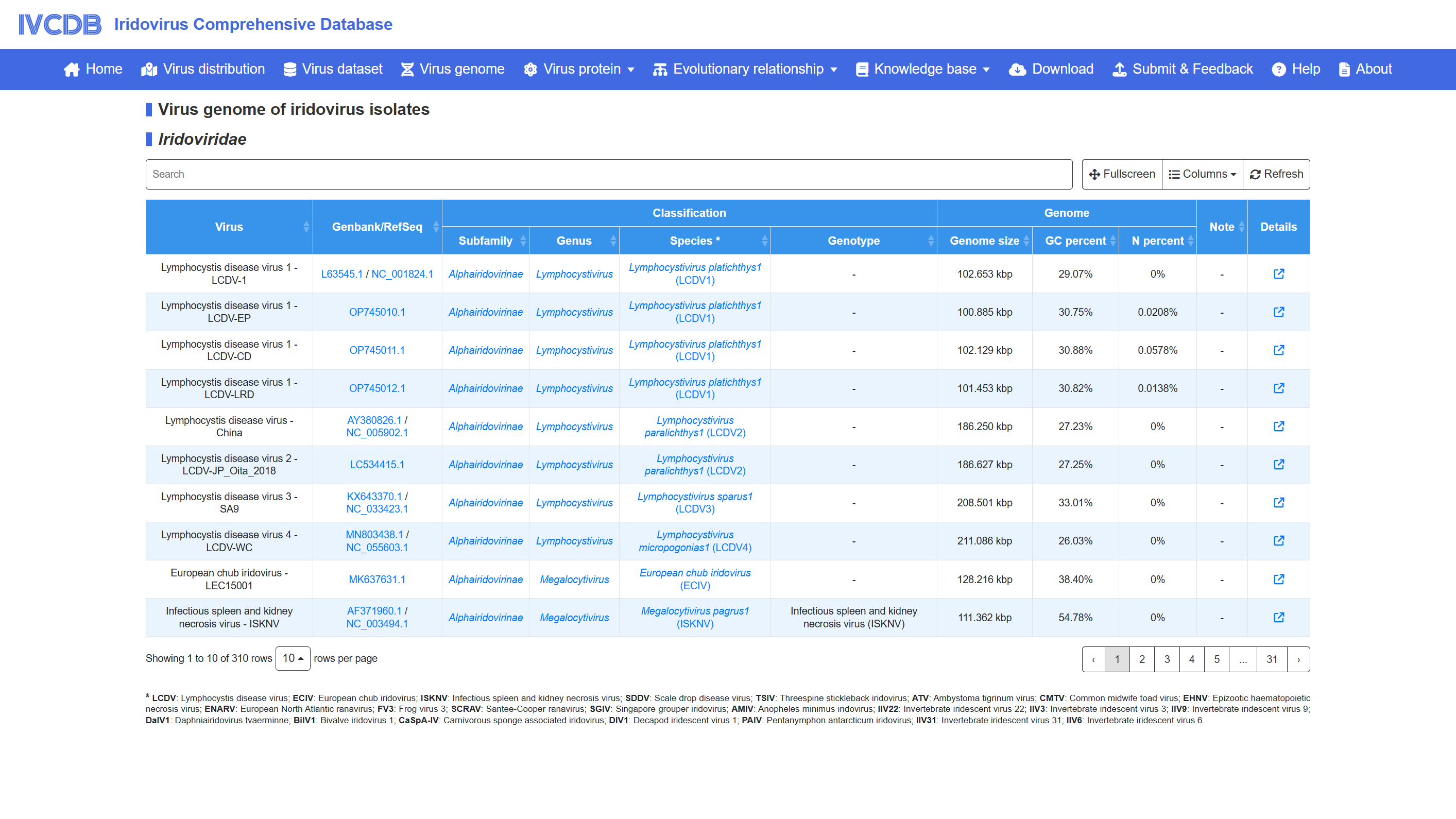


**Supplementary Figure S4.** Navigation interface in tabular form of the virus genome (exemplified by the virus genome of the family *Iridoviridae*).


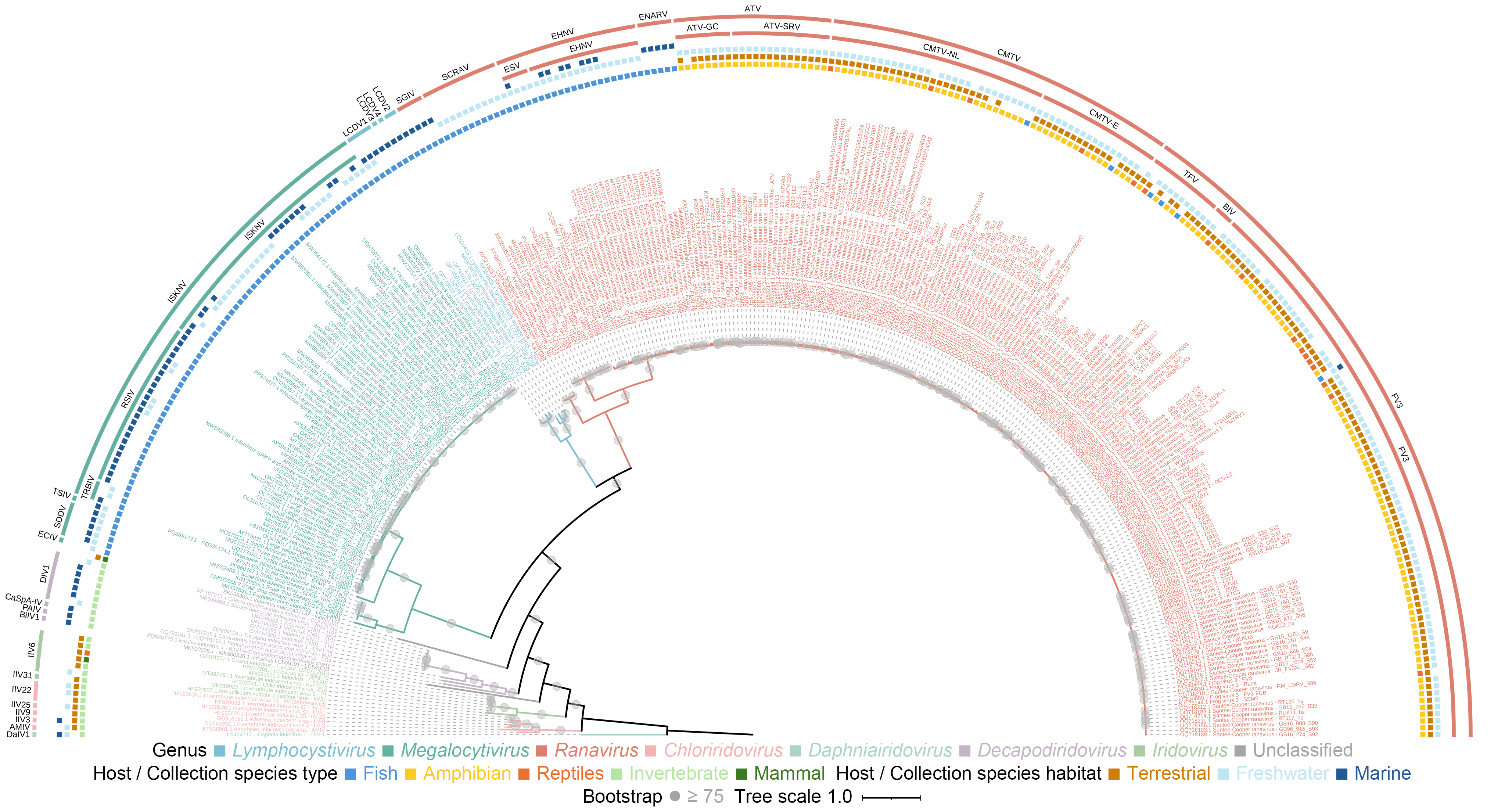


**Supplementary Figure S5.** The phylogenetic evolutionary tree with evolutionary distances of the family *Iridoviridae* based on the core genes/proteins. The colors of the branches and nodes represent the genera, while the outermost colored stripes represent the virus species and genotypes. The colored squares represent the type and habitat of the host or collection species of the virus isolates.


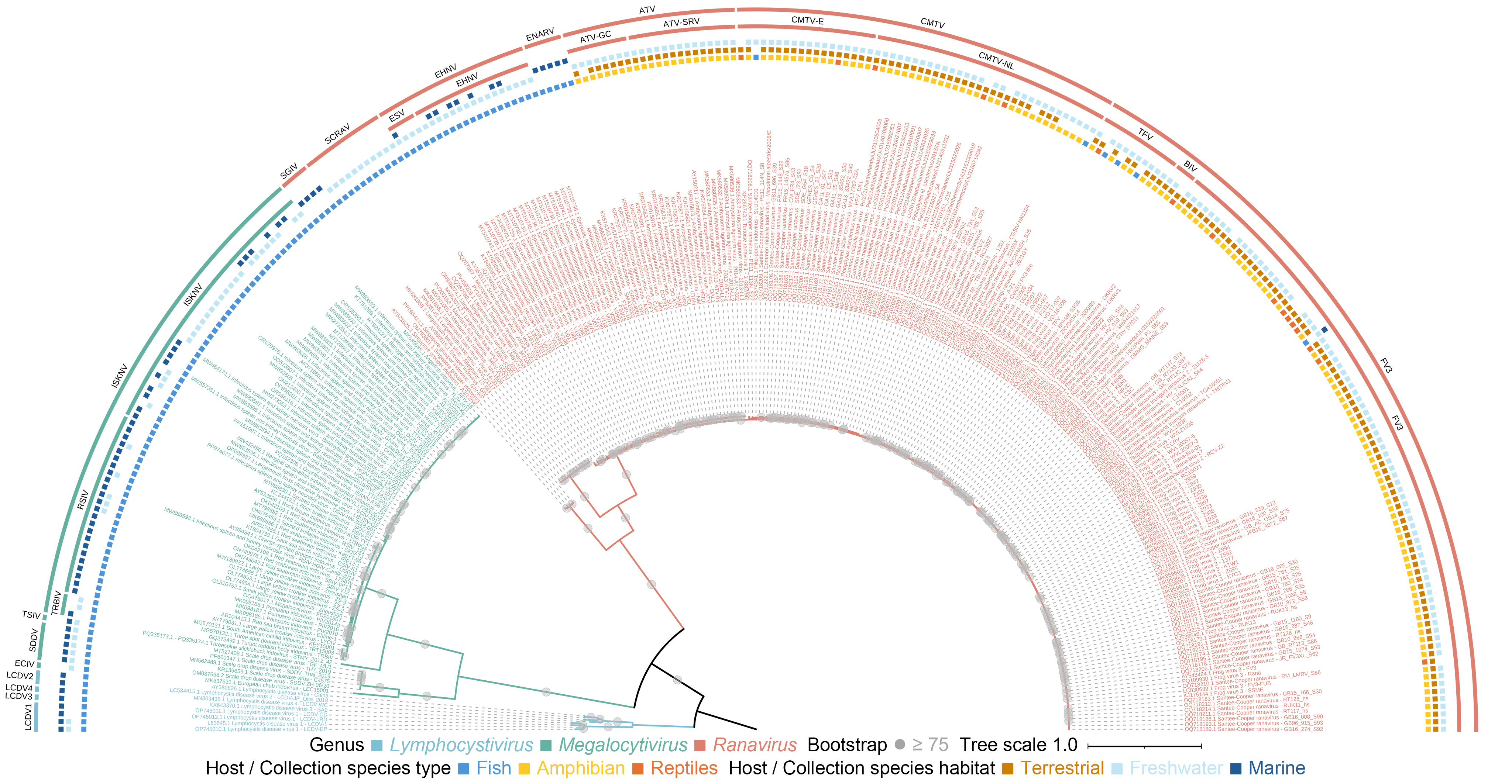


**Supplementary Figure S6.** The phylogenetic evolutionary tree with evolutionary distances of the subfamily *Alphairidovirinae* based on the core genes/proteins. The colors of the branches and nodes represent the genera, while the outermost colored stripes represent the virus species and genotypes. The colored squares represent the type and habitat of the host or collection species of the virus isolates.


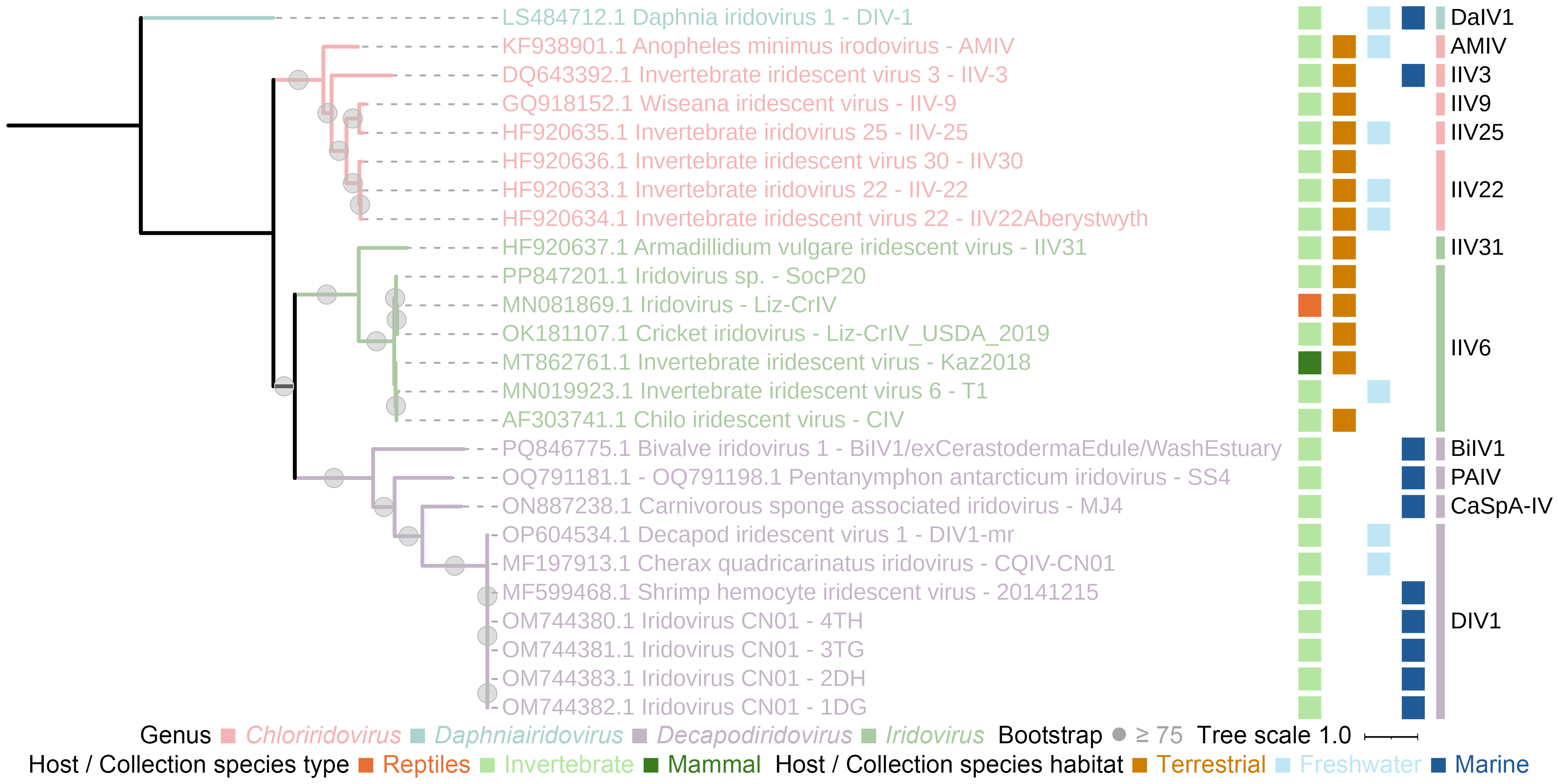


**Supplementary Figure S7.** The phylogenetic evolutionary tree with evolutionary distances of the subfamily *Betairidovirinae* based on the core genes/proteins. The colors of the branches and nodes represent the genera, while the outermost colored stripes represent the virus species. The colored squares represent the type and habitat of the host or collection species of the virus isolates.


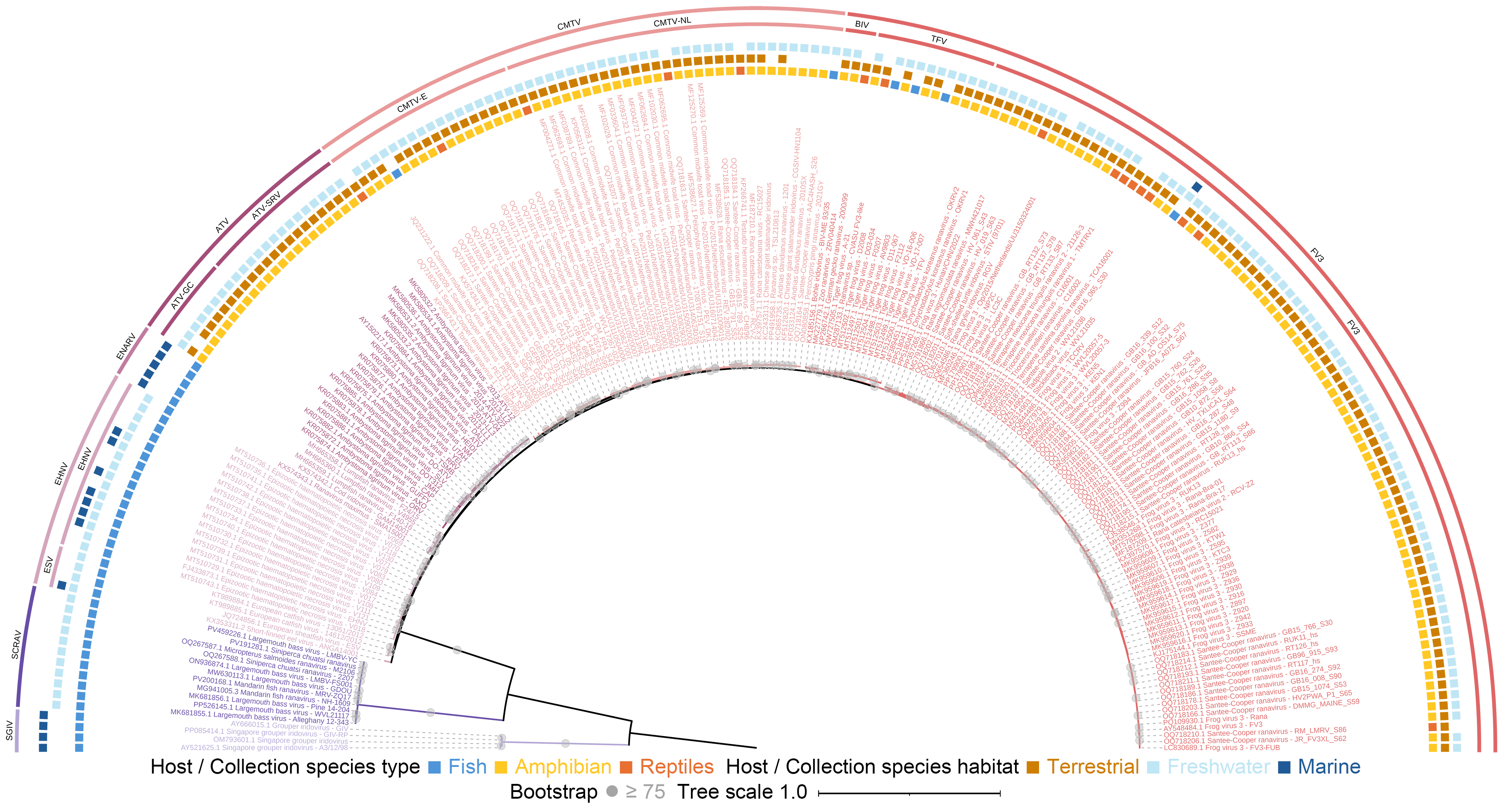


**Supplementary Figure S8.** The phylogenetic evolutionary tree with evolutionary distances of the genus *Ranavirus* based on the core genes/proteins. The colors of the branches and nodes represent the virus species, while the outermost colored stripes represent the virus species and genotypes. The colored squares represent the type and habitat of the host or collection species of the virus isolates.


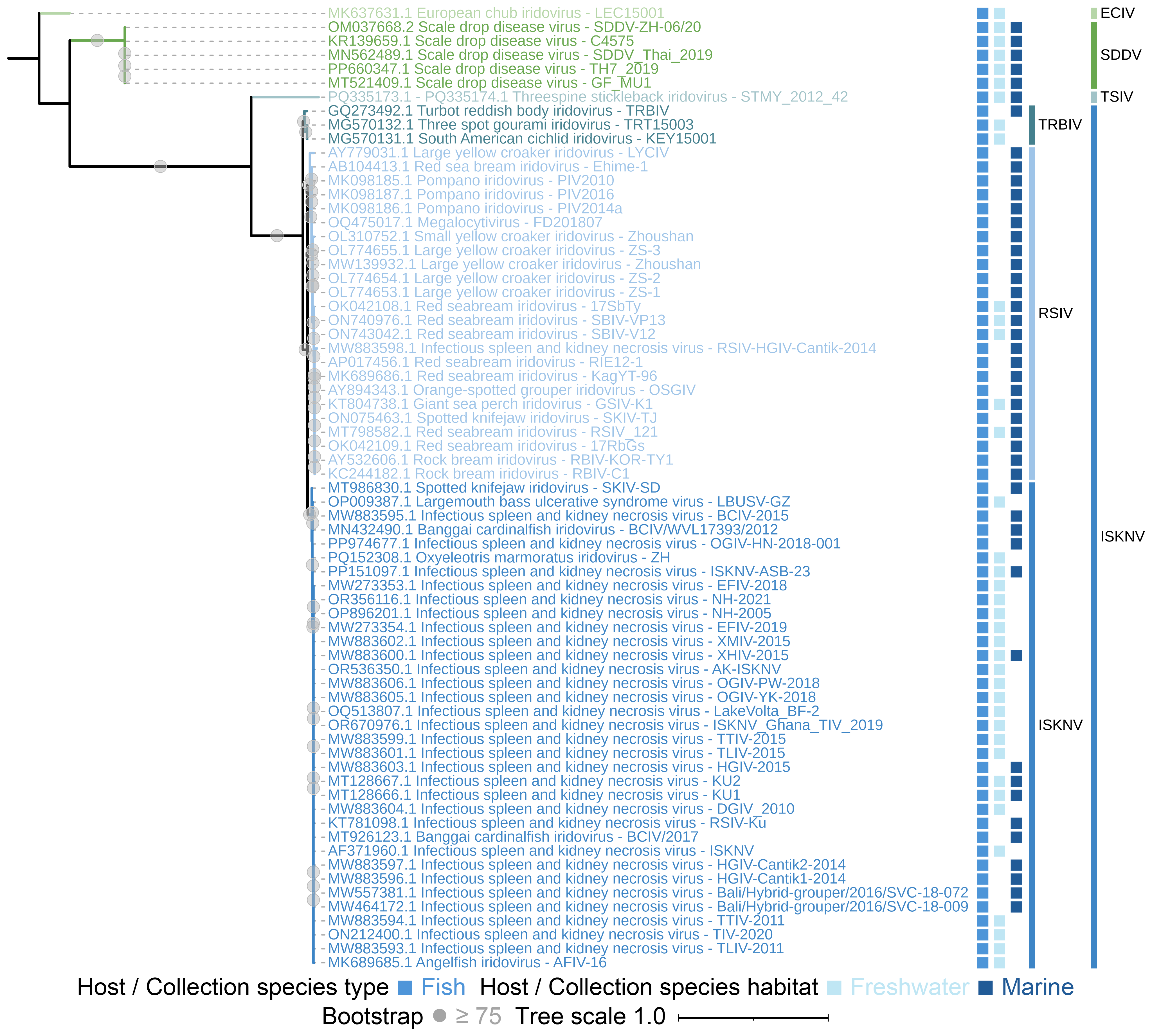


**Supplementary Figure S9.** The phylogenetic evolutionary tree with evolutionary distances of the genus *Megalocytivirus* based on the core genes/proteins. The colors of the branches and nodes represent the virus species, while the outermost colored stripes represent the virus species and genotypes. The colored squares represent the type and habitat of the host or collection species of the virus isolates.


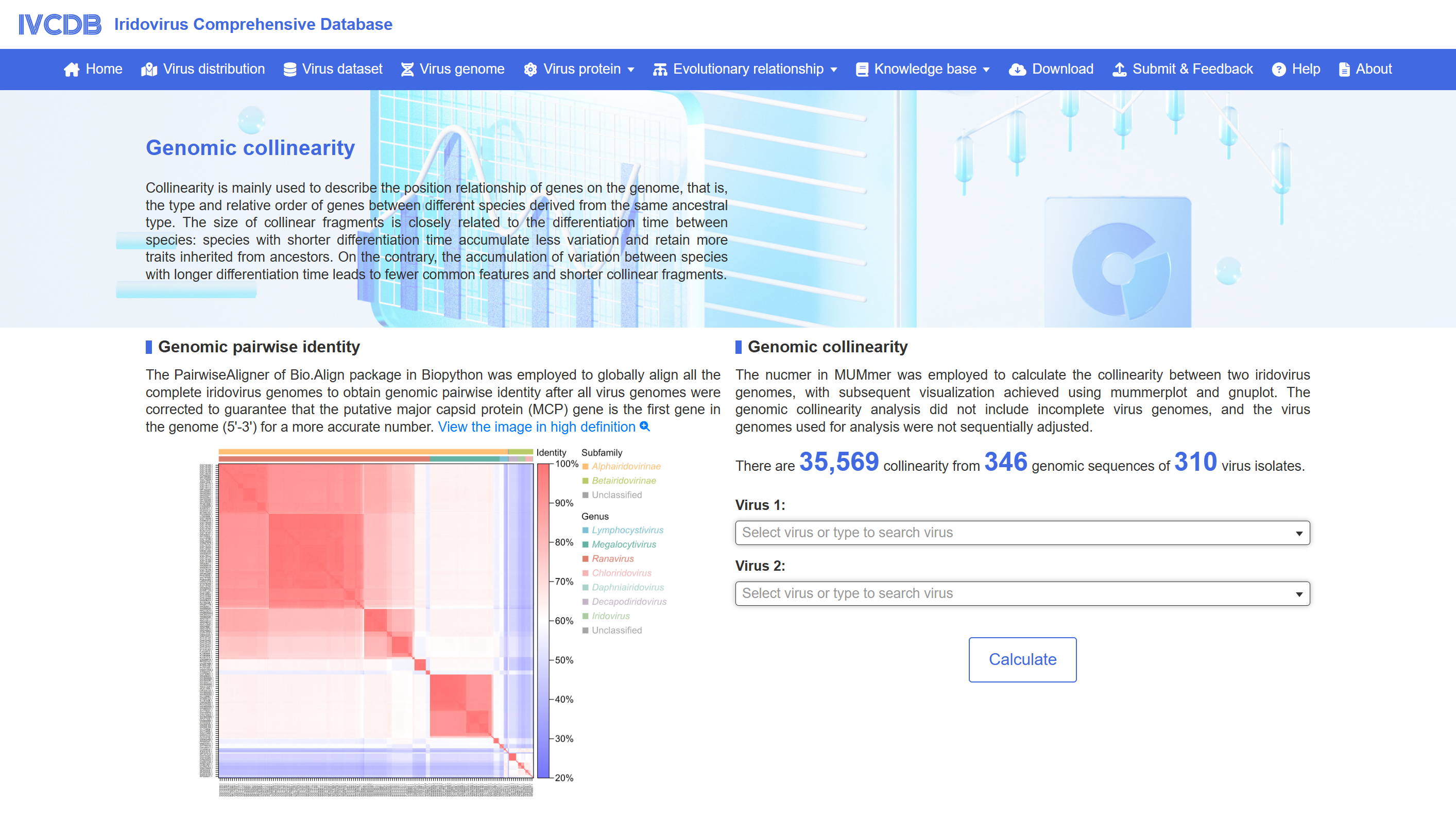


**Supplementary Figure S10.** The genomic collinearity page. The genomic pairwise identity is displayed on the left, while the genome collinearity analysis system is placed on the right.


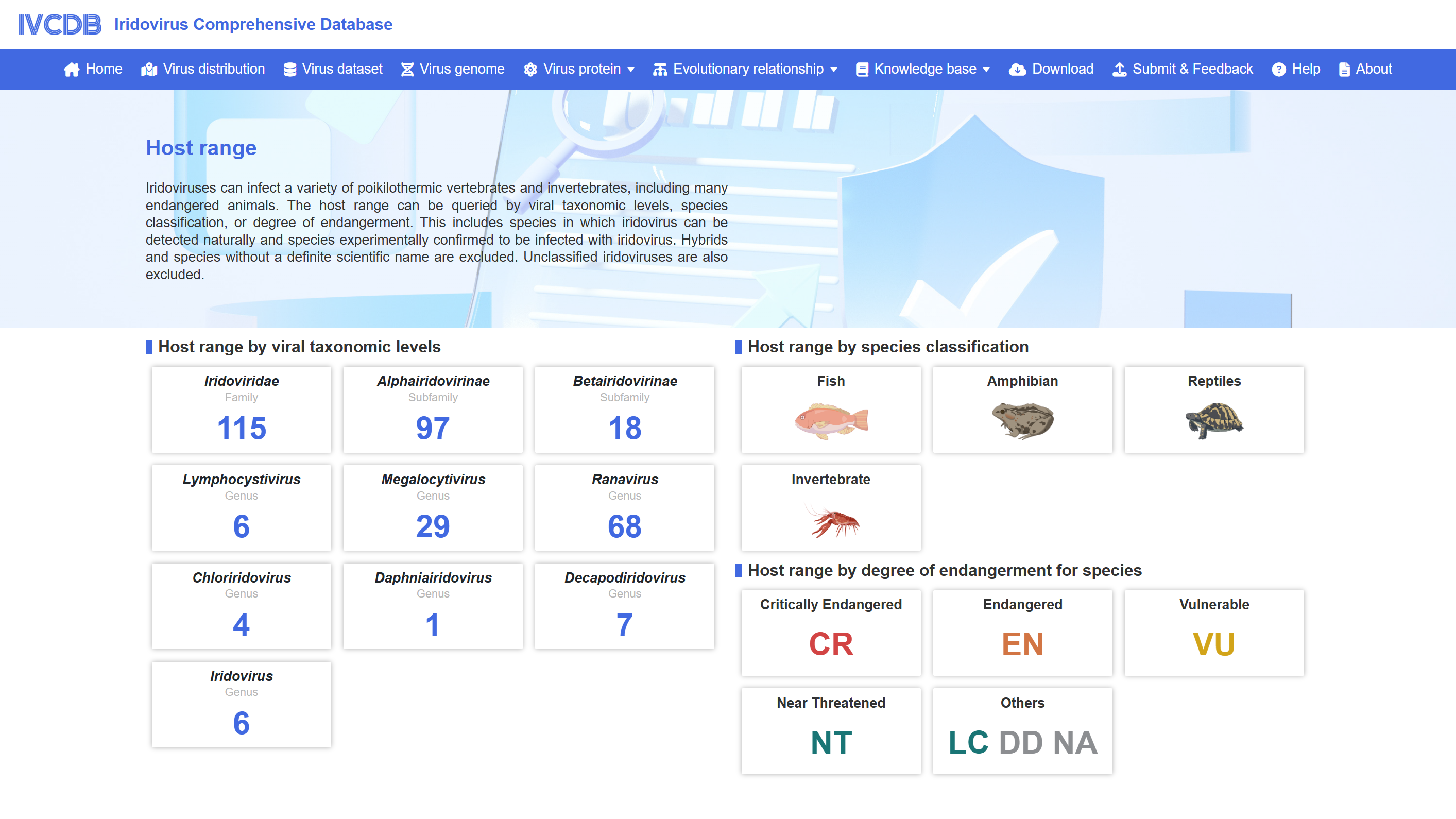


**Supplementary Figure S11.** Card-style navigation interface for the host range data in the application-oriented knowledge base.


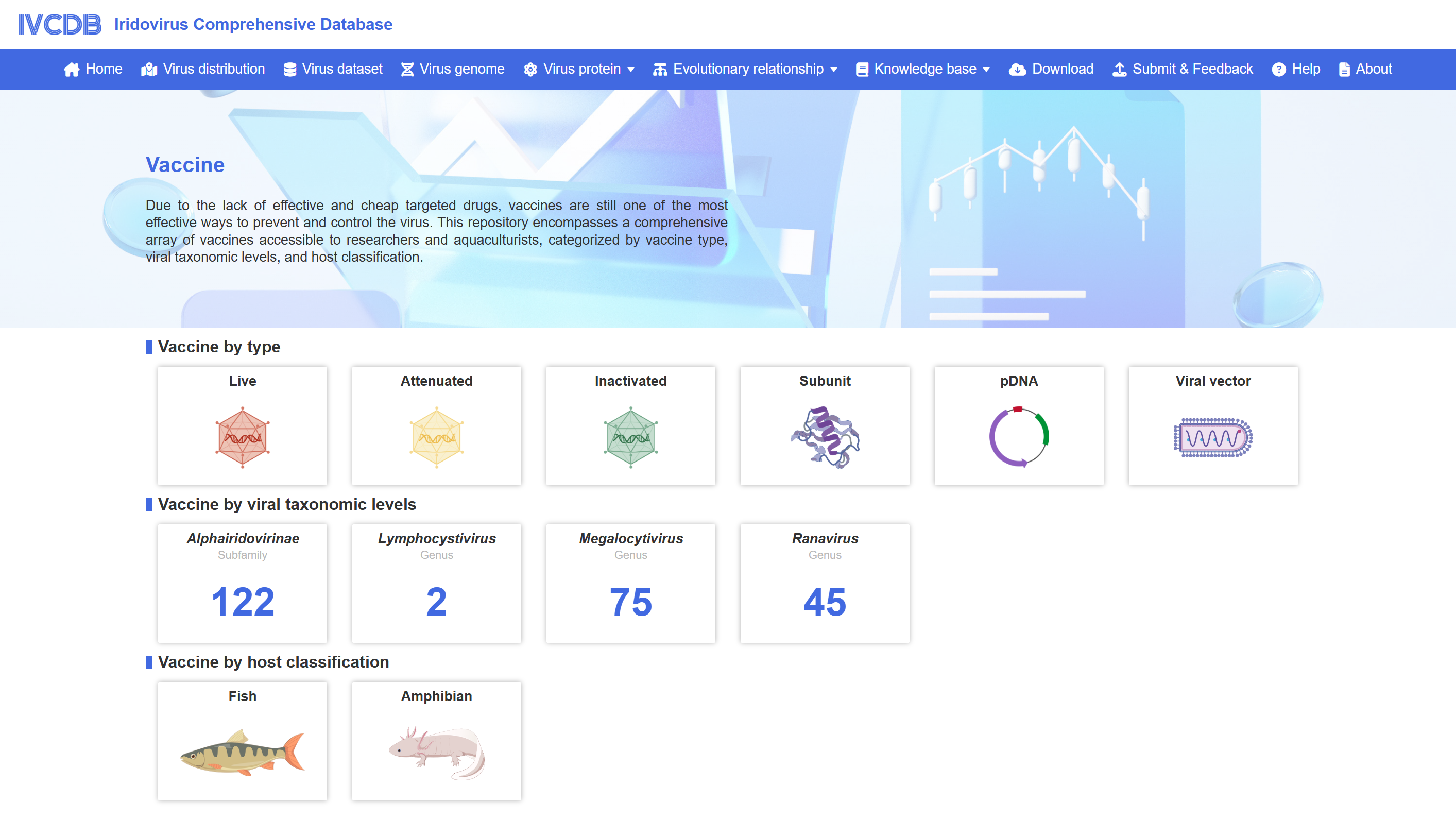


**Supplementary Figure S12.** Card-style navigation interface for the experimentally validated vaccines in the application-oriented knowledge base.


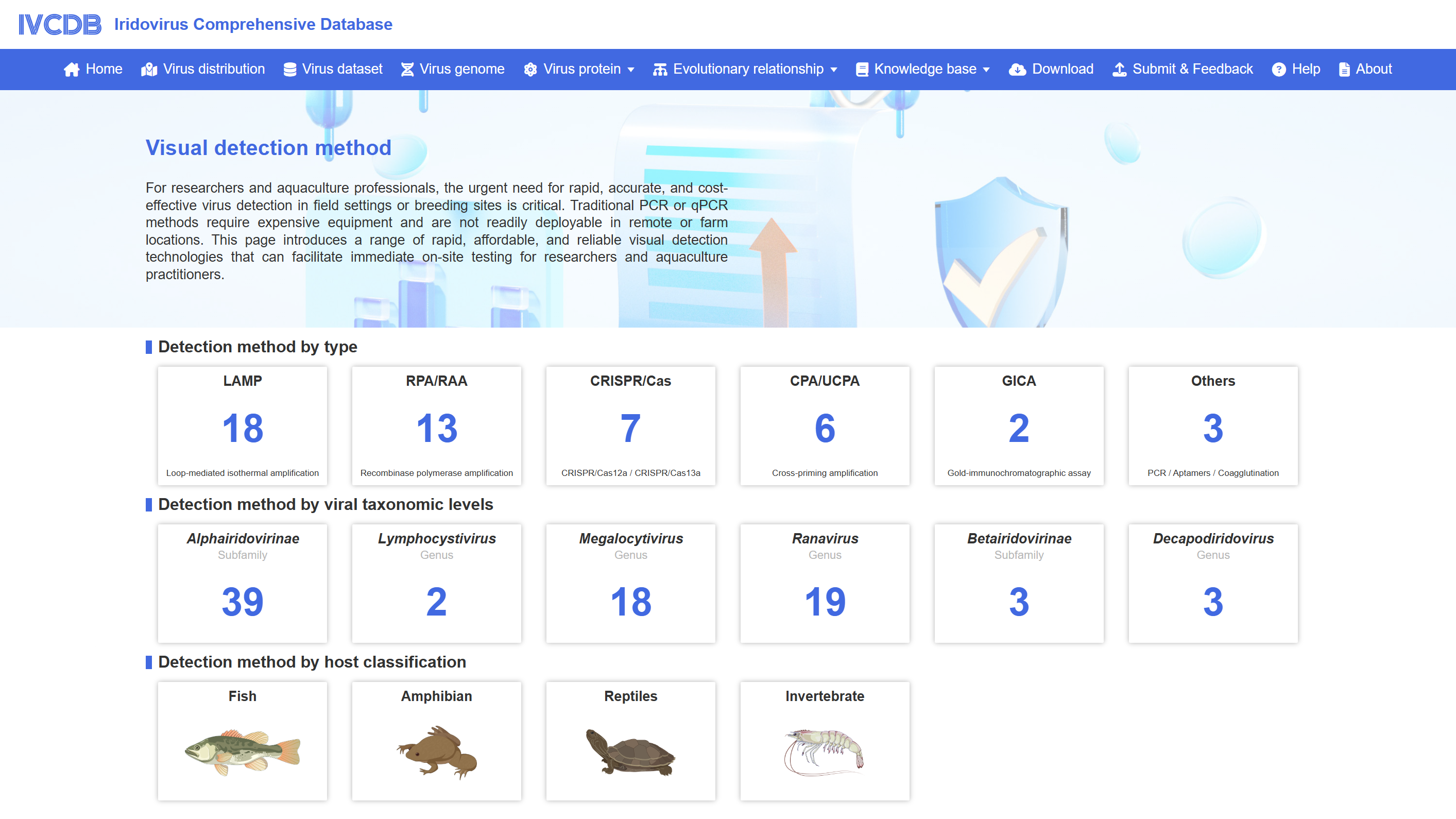


**Supplementary Figure S13.** Card-style navigation interface for the field-deployable visual detection methods in the application-oriented knowledge base.

**Supplementary Table S1.** Virus isolates with corrected taxonomic levels (subfamily and/or genus).

| **Virus isolate** | **Genbank** | **Original taxonomic levels** | | | **Corrected taxonomic levels** | | | **VISTA** | | | | **Note** | **Reference** |
| --- | --- | --- | --- | --- | --- | --- | --- | --- | --- | --- | --- | --- | --- |
|  |  | **Subfamily** | **Genus** | **Species *** | **Subfamily** | **Genus** | **Species *** | **Closest match genus** | **Closest match species *** | **Distance** | **Assignment** |  |  |
| European chub iridovirus - LEC15001 | MK637631.1 | - | - | *European chub iridovirus* (ECIV) | *Alphairidovirinae* | *Megalocytivirus* | *European chub iridovirus* (ECIV) | *Lymphocystivirus* | *Lymphocystivirus platichthys1* (LCDV1) | 0.6812 | Different genera | Possiblenovel virus species | [1] |
| Bivalve iridovirus 1 - BiIV1/  exCerastodermaEdule/  WashEstuary | PQ846775.1 | *Betairidovirinae* | *Iridovirus* | *Bivalve iridovirus 1* (BiIV1) | *Betairidovirinae* | *Decapodiridovirus* | *Bivalve iridovirus 1* (BiIV1) | *Iridovirus* | *Iridovirus armadillidium1* (IIV31) | 0.7532 | Different genera | Possible novel virus species | [2] |
| Carnivorous sponge associated iridovirus - MJ4 | ON887238.1 | *-* | - | *Carnivorous sponge associated iridovirus* (CaSpA-IV) | *Betairidovirinae* | *Decapodiridovirus* | *Carnivorous sponge associated iridovirus* (CaSpA-IV) | *Chloriridovirus* | *Chloriridovirus simulium2* (IIV25) | 0.7206 | Different genera | Possible novel virus species | [3] |
| Pentanymphon antarcticum iridovirus - SS4 | OQ791181.1 - OQ791198.1 | - | *-* | *Pentanymphon antarcticum iridovirus* (PAIV) | *Betairidovirinae* | *Decapodiridovirus* | *Pentanymphon antarcticum iridovirus* (PAIV) | *Lymphocystivirus* ** | *Lymphocystivirus platichthys1* (LCDV1) ** | 0.8126 ** | Different genera ** | Possible novel virus species | [4] |
| Oxyeleotris marmoratus iridovirus - ZH | PQ152308.1 | *Betairidovirinae* | *Iridovirus* | *Oxyeleotris marmoratus iridovirus* | *Alphairidovirinae* | *Megalocytivirus* | *Megalocytivirus pagrus1* (ISKNV) | *Megalocytivirus* | *Megalocytivirus pagrus1* (ISKNV) | 0.2580 | Same genus | - | [5] |
| Cherax quadricarinatus iridovirus -  CQIV-CN01 | MF197913.1 | - | - | *Cherax quadricarinatus iridovirus* | *Betairidovirinae* | *Decapodiridovirus* | *Decapodiridovirus litopenaeus1* (DIV1) | *Chloriridovirus* | *Chloriridovirus anopheles1* (AMIV) | 0.6972 | Different genera | - | [6] |
| Iridovirus CN01 - 4TH | OM744380.1 | *Betairidovirinae* | *Iridovirus* | *Iridovirus CN01* | *Betairidovirinae* | *Decapodiridovirus* | *Decapodiridovirus litopenaeus1* (DIV1) | *Chloriridovirus* | *Chloriridovirus anopheles1* (AMIV) | 0.6945 | Different genera | - | [7] |
| Iridovirus CN01 - 3TG | OM744381.1 | *Betairidovirinae* | *Iridovirus* | *Iridovirus CN01* | *Betairidovirinae* | *Decapodiridovirus* | *Decapodiridovirus litopenaeus1* (DIV1) | *Chloriridovirus* | *Chloriridovirus anopheles1* (AMIV) | 0.6922 | Different genera | - | [7] |
| Iridovirus CN01 - 1DG | OM744382.1 | *Betairidovirinae* | *Iridovirus* | *Iridovirus CN01* | *Betairidovirinae* | *Decapodiridovirus* | *Decapodiridovirus litopenaeus1* (DIV1) | *Chloriridovirus* | *Chloriridovirus anopheles1* (AMIV) | 0.6959 | Different genera | - | [7] |
| Iridovirus CN01 - 2DH | OM744383.1 | *Betairidovirinae* | *Iridovirus* | *Iridovirus CN01* | *Betairidovirinae* | *Decapodiridovirus* | *Decapodiridovirus litopenaeus1* (DIV1) | *Chloriridovirus* | *Chloriridovirus anopheles1* (AMIV) | 0.6958 | Different genera | - | [7] |
| Iridovirus - Liz-CrIV | MN081869.1 | - | - | *Iridovirus Liz-CrIV* | *Betairidovirinae* | *Iridovirus* | *Iridovirus chilo1* (IIV6) | *Iridovirus* | *Iridovirus chilo1* (IIV6) | 0.3916 | Same genus | - | [8] |
| Cricket iridovirus - Liz-CrIV_USDA_2019 | OK181107.1 | - | - | *Cricket iridovirus* | *Betairidovirinae* | *Iridovirus* | *Iridovirus chilo1* (IIV6) | *Iridovirus* | *Iridovirus chilo1* (IIV6) | 0.3879 | Same genus | - | [9] |

***** ECIV: European chub iridovirus; LCDV1: Lymphocystis disease virus 1; BiIV1: Bivalve iridovirus 1; IIV31: Invertebrate iridescent virus 31; CaSpA-IV: Carnivorous sponge associated iridovirus; IIV25: Invertebrate iridescent virus 25; PAIV: Pentanymphon antarcticum iridovirus; ISKNV: Infectious spleen and kidney necrosis virus; DIV1: Decapod iridescent virus 1; AMIV: Anopheles minimus iridovirus; IIV6: Invertebrate iridescent virus 6.

** The analysis based on the longest sequence (OQ791181.1) in the viral genome.

**References**

1. Halaly MA, Subramaniam K, Koda SA *et al*. Characterization of a novel megalocytivirus isolated from European chub (*Squalius cephalus*). *Viruses* 2019;11:440. https://doi.org/10.3390/v11050440
2. Hooper C, Tidy AM, Jessop R *et al*. Genomic and morphological characterisation of a novel iridovirus, bivalve iridovirus 1 (BiIV1), infecting the common cockle (*Cerastoderma edule*). bioRxiv, https://doi.org/10.1101/2025.03.05.641634, 10 March 2025, preprint: not peer reviewed.
3. Canuti M, Large G, Verhoeven JTP *et al*. A novel iridovirus discovered in deep-sea carnivorous sponges. *Viruses* 2022;14:1595. https://doi.org/10.3390/v14081595
4. Bojko J, Maxwell JM, Burgess AL *et al*. An iridovirus from the Antarctic seaspider *Pentanymphon antarcticum* (Pycnogonida). *Antarct Sci* 2024;36:47–50. https://doi.org/10.1017/S0954102024000063
5. Gu Y, Xu Y, Chen X *et al*. Discovery and analysis of Oxyeleotris marmoratus iridovirus isolated from marble goby revealing genome structure and pathogenicity. SSRN, http://dx.doi.org/10.2139/ssrn.5358861, 22 July 2025, preprint: not peer reviewed.
6. Li F, Xu L, Yang F. Genomic characterization of a novel iridovirus from redclaw crayfish *Cherax quadricarinatus*: evidence for a new genus within the family *Iridoviridae*. *J Gen Virol* 2017;98:2589–95. https://doi.org/10.1099/jgv.0.000904
7. Qin P, Wang G, Luan Y *et al*. Complete genome sequence and characterization of four Decapod iridescent virus 1 isolates from crab and shrimp. *J Invertebr Pathol* 2023;196:107852. https://doi.org/10.1016/j.jip.2022.107852
8. Papp T, Marschang RE. Detection and characterization of invertebrate iridoviruses found in reptiles and prey insects in Europe over the past two decades. *Viruses* 2019;11:600. https://doi.org/10.3390/v11070600
9. Duffield KR, Hunt J, Sadd BM *et al*. Active and covert infections of cricket iridovirus and *Acheta domesticus* densovirus in reared *Gryllodes* sigillatus crickets. *Front Microbiol* 2021;12:780796. https://doi.org/10.3389/fmicb.2021.780796

**Supplementary Table S2.** Virus isolates with corrected taxonomic level (species).

| **Virus isolate** | **Genbank** | **Subfamily** | **Genus** | **Original species *** | **Corrected species *** | **VISTA** | | | | **Note** | **Reference** |
| --- | --- | --- | --- | --- | --- | --- | --- | --- | --- | --- | --- |
|  |  |  |  |  |  | **Closest match genus** | **Closest match species *** | **Distance** | **Assignment** |  |  |
| Threespine stickleback iridovirus - STMY_2012_42 | PQ335173.1 - PQ335174.1 | *Alphairidovirinae* | *Megalocytivirus* | *Megalocytivirus pagrus1* (ISKNV) | *Threespine stickleback iridovirus* (TSIV) | *Ranavirus* ** | Ranavirus epinephelus1 (SGIV) ** | 0.7379 ** | Different genera ** | Possible novel virus species | [1] |
| Small yellow croaker iridovirus - Zhoushan | OL310752.1 | *Alphairidovirinae* | *Megalocytivirus* | *Small yellow croaker iridovirus* | *Megalocytivirus pagrus1* (ISKNV) | *Megalocytivirus* | *Megalocytivirus pagrus1* (ISKNV) | 0.3450 | Same genus | - | [2] |
| Megalocytivirus - FD201807 | OQ475017.1 | *Alphairidovirinae* | *Megalocytivirus* | *Megalocytivirus FD201807* | *Megalocytivirus pagrus1* (ISKNV) | *Megalocytivirus* | *Megalocytivirus pagrus1* (ISKNV) | 0.3566 | Same genus | - | [3] |
| South American cichlid iridovirus - KEY15001 | MG570131.1 | *Alphairidovirinae* | *Megalocytivirus* | *South American cichlid iridovirus* | *Megalocytivirus pagrus1* (ISKNV) | *Megalocytivirus* | *Megalocytivirus pagrus1* (ISKNV) | 0.5228 | Same genus | - | [4] |
| Three spot gourami iridovirus - TRT15003 | MG570132.1 | *Alphairidovirinae* | *Megalocytivirus* | *Three spot gourami iridovirus* | *Megalocytivirus pagrus1* (ISKNV) | *Megalocytivirus* | *Megalocytivirus pagrus1* (ISKNV) | 0.5249 | Same genus | - | [4] |
| Tortoise ranavirus 1 - 1 (882/96) | KP266743.1 | *Alphairidovirinae* | *Ranavirus* | *Ranavirus rana1* (FV3) | *Ranavirus alytes1* (CMTV) | *Ranavirus* | *Ranavirus alytes1* (CMTV) | 0.0000 | Same species | - | [5] |
| Santee-Cooper ranavirus - CM_FR4_S43 | OQ718165.1 | *Alphairidovirinae* | *Ranavirus* | *Ranavirus micropterus1* (SCRAV) | *Ranavirus alytes1* (CMTV) | *Ranavirus* | *Ranavirus alytes1* (CMTV) | 0.1522 | Same species | - | - |
| Santee-Cooper ranavirus - FR15_1448_S22 | OQ718167.1 | *Alphairidovirinae* | *Ranavirus* | *Ranavirus micropterus1* (SCRAV) | *Ranavirus alytes1* (CMTV) | *Ranavirus* | *Ranavirus alytes1* (CMTV) | 0.1484 | Same species | - | - |
| Santee-Cooper ranavirus - FR15_1457a_S95 | OQ718168.1 | *Alphairidovirinae* | *Ranavirus* | *Ranavirus micropterus1* (SCRAV) | *Ranavirus alytes1* (CMTV) | *Ranavirus* | *Ranavirus alytes1* (CMTV) | 0.1509 | Same species | - | - |
| Santee-Cooper ranavirus - GA10_C5_S33 | OQ718169.1 | *Alphairidovirinae* | *Ranavirus* | *Ranavirus micropterus1* (SCRAV) | *Ranavirus alytes1* (CMTV) | *Ranavirus* | *Ranavirus rana1* (FV3) | 0.1665 | Same species | - | - |
| Santee-Cooper ranavirus - GA11_01_S47 | OQ718170.1 | *Alphairidovirinae* | *Ranavirus* | *Ranavirus micropterus1* (SCRAV) | *Ranavirus alytes1* (CMTV) | *Ranavirus* | *Ranavirus rana1* (FV3) | 0.1704 | Same species | - | - |
| Santee-Cooper ranavirus - GA11_05_S46 | OQ718171.1 | *Alphairidovirinae* | *Ranavirus* | *Ranavirus micropterus1* (SCRAV) | *Ranavirus alytes1* (CMTV) | *Ranavirus* | *Ranavirus rana1* (FV3) | 0.1618 | Same species | - | - |
| Santee-Cooper ranavirus - GA13_334S2_S40 | OQ718172.1 | *Alphairidovirinae* | *Ranavirus* | *Ranavirus micropterus1* (SCRAV) | *Ranavirus alytes1* (CMTV) | *Ranavirus* | *Ranavirus rana1* (FV3) | 0.1617 | Same species | - | - |
| Santee-Cooper ranavirus - GA13_354S2_S50 | OQ718173.1 | *Alphairidovirinae* | *Ranavirus* | *Ranavirus micropterus1* (SCRAV) | *Ranavirus alytes1* (CMTV) | *Ranavirus* | *Ranavirus rana1* (FV3) | 0.1585 | Same species | - | - |
| Santee-Cooper ranavirus - GB11_696_S39 | OQ718176.1 | *Alphairidovirinae* | *Ranavirus* | *Ranavirus micropterus1* (SCRAV) | *Ranavirus alytes1* (CMTV) | *Ranavirus* | *Ranavirus rana1* (FV3) | 0.1907 | Same species | - | - |
| Santee-Cooper ranavirus - GERES_22_S20 | OQ718199.1 | *Alphairidovirinae* | *Ranavirus* | *Ranavirus micropterus1* (SCRAV) | *Ranavirus alytes1* (CMTV) | *Ranavirus* | *Ranavirus alytes1* (CMTV) | 0.2020 | Same species | - | - |
| Santee-Cooper ranavirus - GERES_C5_S4 | OQ718200.1 | *Alphairidovirinae* | *Ranavirus* | *Ranavirus micropterus1* (SCRAV) | *Ranavirus alytes1* (CMTV) | *Ranavirus* | *Ranavirus alytes1* (CMTV) | 0.1973 | Same species | - | - |
| Santee-Cooper ranavirus - PE11_113N_S27 | OQ718208.1 | *Alphairidovirinae* | *Ranavirus* | *Ranavirus micropterus1* (SCRAV) | *Ranavirus alytes1* (CMTV) | *Ranavirus* | *Ranavirus rana1* (FV3) | 0.1830 | Same species | - | - |
| Santee-Cooper ranavirus - PE11_114N_S8 | OQ718209.1 | *Alphairidovirinae* | *Ranavirus* | *Ranavirus micropterus1* (SCRAV) | *Ranavirus alytes1* (CMTV) | *Ranavirus* | *Ranavirus alytes1* (CMTV) | 0.0269 | Same species | - | - |
| Santee-Cooper ranavirus - SDE_G54_S18 | OQ718216.1 | *Alphairidovirinae* | *Ranavirus* | *Ranavirus micropterus1* (SCRAV) | *Ranavirus alytes1* (CMTV) | *Ranavirus* | *Ranavirus alytes1* (CMTV) | 0.1756 | Same species | - | - |
| Santee-Cooper ranavirus - SDE_I23_S87 | OQ718217.1 | *Alphairidovirinae* | *Ranavirus* | *Ranavirus micropterus1* (SCRAV) | *Ranavirus alytes1* (CMTV) | *Ranavirus* | *Ranavirus alytes1* (CMTV) | 0.1765 | Same species | - | - |
| Chinese giant salamander iridovirus | KC243313.1 | *Alphairidovirinae* | *Ranavirus* | *Ranavirus rana1* (FV3) | *Ranavirus alytes1* (CMTV) | *Ranavirus* | *Ranavirus rana1* (FV3) | 0.0000 | Same species | - | - |
| Chinese giant salamander iridovirus - CGSIV-HN1104 | KF512820.1 | *Alphairidovirinae* | *Ranavirus* | *Ranavirus rana1* (FV3) | *Ranavirus alytes1* (CMTV) | *Ranavirus* | *Ranavirus alytes1* (CMTV) | 0.0494 | Same species | - | [6] |
| Rana catesbeiana virus - RC15027 | KX397571.1 | *Alphairidovirinae* | *Ranavirus* | *Rana catesbeiana virus* | *Ranavirus alytes1* (CMTV) | *Ranavirus* | *Ranavirus alytes1* (CMTV) | 0.1050 | Same species | - | - |
| Rana catesbeiana virus - RCV-Z | MF187210.1 | *Alphairidovirinae* | *Ranavirus* | *Rana catesbeiana virus* | *Ranavirus alytes1* (CMTV) | *Ranavirus* | *Ranavirus alytes1* (CMTV) | 0.1050 | Same species | - | [7] |
| Red-eared slider ranavirus - WVL17367-02A | MT452035.1 | *Alphairidovirinae* | *Ranavirus* | *Red-eared slider ranavirus* | *Ranavirus alytes1* (CMTV) | *Ranavirus* | *Ranavirus alytes1* (CMTV) | 0.1585 | Same species | - | [8] |
| Percocypris pingi ranavirus - 2021GY | ON080858.1 | *Alphairidovirinae* | *Ranavirus* | *Percocypris pingi ranavirus* | *Ranavirus alytes1* (CMTV) | *Ranavirus* | *Ranavirus alytes1* (CMTV) | 0.0252 | Same species | - | [9] |
| Santee-Cooper ranavirus - 1708T67151_S15 | OQ718163.1 | *Alphairidovirinae* | *Ranavirus* | *Ranavirus micropterus1* (SCRAV) | *Ranavirus alytes1* (CMTV) | *Ranavirus* | *Ranavirus alytes1* (CMTV) | 0.1378 | Same species | - | - |
| Santee-Cooper ranavirus - AAC4HASH_S26 | OQ718164.1 | *Alphairidovirinae* | *Ranavirus* | *Ranavirus micropterus1* (SCRAV) | *Ranavirus alytes1* (CMTV) | *Ranavirus* | *Ranavirus alytes1* (CMTV) | 0.0437 | Same species | - | - |
| Santee-Cooper ranavirus - GB15_789_S25 | OQ718184.1 | *Alphairidovirinae* | *Ranavirus* | *Ranavirus micropterus1* (SCRAV) | *Ranavirus alytes1* (CMTV) | *Ranavirus* | *Ranavirus alytes1* (CMTV) | 0.0627 | Same species | - | - |
| Santee-Cooper ranavirus - GB15_791_S52 | OQ718185.1 | *Alphairidovirinae* | *Ranavirus* | *Ranavirus micropterus1* (SCRAV) | *Ranavirus alytes1* (CMTV) | *Ranavirus* | *Ranavirus alytes1* (CMTV) | 0.0615 | Same species | - | - |
| Santee-Cooper ranavirus - NL3170726027_S4 | OQ718207.1 | *Alphairidovirinae* | *Ranavirus* | *Ranavirus micropterus1* (SCRAV) | *Ranavirus alytes1* (CMTV) | *Ranavirus* | *Ranavirus alytes1* (CMTV) | 0.0234 | Same species | - | - |
| Ranavirus sp. - TSL210813 | PP491958.1 | *Alphairidovirinae* | *Ranavirus* | *Ranavirus* sp. | *Ranavirus alytes1* (CMTV) | *Ranavirus* | *Ranavirus alytes1* (CMTV) | 0.0823 | Same species | - | [10] |
| Short-finned eel virus - ANGA14001 | KX353311.2 | *Alphairidovirinae* | *Ranavirus* | *Ranavirus rana1* (FV3) | *Ranavirus perca1* (EHNV) | *Ranavirus* | *Short-finned eel ranavirus* | 0.0000 | Same species | - | [11] |
| Cod iridovirus - GAM14001 | KX574342.1 | *Alphairidovirinae* | *Ranavirus* | *Ranavirus rana1* (FV3) | *Ranavirus gadus1* (ENARV) | *Ranavirus* | *Ranavirus rana1* (FV3) | 0.1074 | Same species | - | [12] |
| Ranavirus maximus - SMA15001 | KX574343.1 | *Alphairidovirinae* | *Ranavirus* | *Ranavirus rana1* (FV3) | *Ranavirus gadus1* (ENARV) | *Ranavirus* | *Ranavirus rana1* (FV3) | 0.0000 | Same species | - | [12] |
| Rana catesbeiana virus 2 - RCV-Z2 | MF187209.1 | *Alphairidovirinae* | *Ranavirus* | *Rana catesbeiana virus 2* | *Ranavirus rana1* (FV3) | *Ranavirus* | *Ranavirus rana1* (FV3) | 0.0000 | Same species | - | [7] |
| Stickleback virus - WVL21035 | MZ514903.1 | *Alphairidovirinae* | *Ranavirus* | *Stickleback virus* | *Ranavirus rana1* (FV3) | *Ranavirus* | *Ranavirus rana1* (FV3) | 0.0820 | Same species | - | [13] |
| Tadpole virus 2 - WVL21036 | MZ514904.1 | *Alphairidovirinae* | *Ranavirus* | *Tadpole virus 2* | *Ranavirus rana1* (FV3) | *Ranavirus* | *Ranavirus rana1* (FV3) | 0.0723 | Same species | - | [13] |
| Terrapene mexicana triunguis ranavirus 1 - TMTRV1 | OM963013.1 | *Alphairidovirinae* | *Ranavirus* | *Terrapene mexicana triunguis ranavirus 1* | *Ranavirus rana1* (FV3) | *Ranavirus* | *Ranavirus rana1* (FV3) | 0.0694 | Same species | - | [14] |
| Terrapene mexicana triunguis ranavirus 2 - 21126-3 | OP852645.1 | *Alphairidovirinae* | *Ranavirus* | *Terrapene mexicana triunguis ranavirus 2* | *Ranavirus rana1* (FV3) | *Ranavirus* | *Ranavirus rana1* (FV3) | 0.0575 | Same species | - | [14] |
| Santee-Cooper ranavirus - DMMG_MAINE_S59 | OQ718166.1 | *Alphairidovirinae* | *Ranavirus* | *Ranavirus micropterus1* (SCRAV) | *Ranavirus rana1* (FV3) | *Ranavirus* | *Ranavirus rana1* (FV3) | 0.0298 | Same species | - | - |
| Santee-Cooper ranavirus - GB10_866_S54 | OQ718174.1 | *Alphairidovirinae* | *Ranavirus* | *Ranavirus micropterus1* (SCRAV) | *Ranavirus rana1* (FV3) | *Ranavirus* | *Ranavirus rana1* (FV3) | 0.0555 | Same species | - | - |
| Santee-Cooper ranavirus - GB10_872_S56 | OQ718175.1 | *Alphairidovirinae* | *Ranavirus* | *Ranavirus micropterus1* (SCRAV) | *Ranavirus rana1* (FV3) | *Ranavirus* | *Ranavirus rana1* (FV3) | 0.0778 | Same species | - | - |
| Santee-Cooper ranavirus - GB15_1058_S8 | OQ718177.1 | *Alphairidovirinae* | *Ranavirus* | *Ranavirus micropterus1* (SCRAV) | *Ranavirus rana1* (FV3) | *Ranavirus* | *Ranavirus rana1* (FV3) | 0.0612 | Same species | - | - |
| Santee-Cooper ranavirus - GB15_1074_S53 | OQ718178.1 | *Alphairidovirinae* | *Ranavirus* | *Ranavirus micropterus1* (SCRAV) | *Ranavirus rana1* (FV3) | *Ranavirus* | *Ranavirus rana1* (FV3) | 0.0934 | Same species | - | - |
| Santee-Cooper ranavirus - GB15_1180_S9 | OQ718179.1 | *Alphairidovirinae* | *Ranavirus* | *Ranavirus micropterus1* (SCRAV) | *Ranavirus rana1* (FV3) | *Ranavirus* | *Ranavirus rana1* (FV3) | 0.0511 | Same species | - | - |
| Santee-Cooper ranavirus - GB15_760_S24 | OQ718180.1 | *Alphairidovirinae* | *Ranavirus* | *Ranavirus micropterus1* (SCRAV) | *Ranavirus rana1* (FV3) | *Ranavirus* | *Ranavirus rana1* (FV3) | 0.0720 | Same species | - | - |
| Santee-Cooper ranavirus - GB15_761_S25 | OQ718181.1 | *Alphairidovirinae* | *Ranavirus* | *Ranavirus micropterus1* (SCRAV) | *Ranavirus rana1* (FV3) | *Ranavirus* | *Ranavirus rana1* (FV3) | 0.0687 | Same species | - | - |
| Santee-Cooper ranavirus - GB15_762_S26 | OQ718182.1 | *Alphairidovirinae* | *Ranavirus* | *Ranavirus micropterus1* (SCRAV) | *Ranavirus rana1* (FV3) | *Ranavirus* | *Ranavirus rana1* (FV3) | 0.0675 | Same species | - | - |
| Santee-Cooper ranavirus - GB15_766_S30 | OQ718183.1 | *Alphairidovirinae* | *Ranavirus* | *Ranavirus micropterus1* (SCRAV) | *Ranavirus rana1* (FV3) | *Ranavirus* | *Ranavirus rana1* (FV3) | 0.1017 | Same species | - | - |
| Santee-Cooper ranavirus - GB16_008_S90 | OQ718186.1 | *Alphairidovirinae* | *Ranavirus* | *Ranavirus micropterus1* (SCRAV) | *Ranavirus rana1* (FV3) | *Ranavirus* | *Ranavirus rana1* (FV3) | 0.0357 | Same species | - | - |
| Santee-Cooper ranavirus - GB16_065_S30 | OQ718187.1 | *Alphairidovirinae* | *Ranavirus* | *Ranavirus micropterus1* (SCRAV) | *Ranavirus rana1* (FV3) | *Ranavirus* | *Ranavirus rana1* (FV3) | 0.0478 | Same species | - | - |
| Santee-Cooper ranavirus - GB16_100_S32 | OQ718188.1 | *Alphairidovirinae* | *Ranavirus* | *Ranavirus micropterus1* (SCRAV) | *Ranavirus rana1* (FV3) | *Ranavirus* | *Ranavirus rana1* (FV3) | 0.0242 | Same species | - | - |
| Santee-Cooper ranavirus - GB16_274_S92 | OQ718189.1 | *Alphairidovirinae* | *Ranavirus* | *Ranavirus micropterus1* (SCRAV) | *Ranavirus rana1* (FV3) | *Ranavirus* | *Ranavirus rana1* (FV3) | 0.0398 | Same species | - | - |
| Santee-Cooper ranavirus - GB16_286_S35 | OQ718190.1 | *Alphairidovirinae* | *Ranavirus* | *Ranavirus micropterus1* (SCRAV) | *Ranavirus rana1* (FV3) | *Ranavirus* | *Ranavirus rana1* (FV3) | 0.0633 | Same species | - | - |
| Santee-Cooper ranavirus - GB16_287_S48 | OQ718191.1 | *Alphairidovirinae* | *Ranavirus* | *Ranavirus micropterus1* (SCRAV) | *Ranavirus rana1* (FV3) | *Ranavirus* | *Ranavirus rana1* (FV3) | 0.0430 | Same species | - | - |
| Santee-Cooper ranavirus - GB16_339_S12 | OQ718192.1 | *Alphairidovirinae* | *Ranavirus* | *Ranavirus micropterus1* (SCRAV) | *Ranavirus rana1* (FV3) | *Ranavirus* | *Ranavirus rana1* (FV3) | 0.1065 | Same species | - | - |
| Santee-Cooper ranavirus - GB96_915_S93 | OQ718193.1 | *Alphairidovirinae* | *Ranavirus* | *Ranavirus micropterus1* (SCRAV) | *Ranavirus rana1* (FV3) | *Ranavirus* | *Ranavirus rana1* (FV3) | 0.0524 | Same species | - | - |
| Santee-Cooper ranavirus - GB_AD_OS14_S75 | OQ718194.1 | *Alphairidovirinae* | *Ranavirus* | *Ranavirus micropterus1* (SCRAV) | *Ranavirus rana1* (FV3) | *Ranavirus* | *Ranavirus rana1* (FV3) | 0.0273 | Same species | - | - |
| Santee-Cooper ranavirus - GB_RT113_S86 | OQ718195.1 | *Alphairidovirinae* | *Ranavirus* | *Ranavirus micropterus1* (SCRAV) | *Ranavirus rana1* (FV3) | *Ranavirus* | *Ranavirus rana1* (FV3) | 0.0547 | Same species | - | - |
| Santee-Cooper ranavirus - GB_RT132_S73 | OQ718196.1 | *Alphairidovirinae* | *Ranavirus* | *Ranavirus micropterus1* (SCRAV) | *Ranavirus rana1* (FV3) | *Ranavirus* | *Ranavirus rana1* (FV3) | 0.0771 | Same species | - | - |
| Santee-Cooper ranavirus - GB_RT133_S87 | OQ718197.1 | *Alphairidovirinae* | *Ranavirus* | *Ranavirus micropterus1* (SCRAV) | *Ranavirus rana1* (FV3) | *Ranavirus* | *Ranavirus rana1* (FV3) | 0.0676 | Same species | - | - |
| Santee-Cooper ranavirus - GB_RT137_S78 | OQ718198.1 | *Alphairidovirinae* | *Ranavirus* | *Ranavirus micropterus1* (SCRAV) | *Ranavirus rana1* (FV3) | *Ranavirus* | *Ranavirus rana1* (FV3) | 0.0701 | Same species | - | - |
| Santee-Cooper ranavirus - HV_019_S63 | OQ718201.1 | *Alphairidovirinae* | *Ranavirus* | *Ranavirus micropterus1* (SCRAV) | *Ranavirus rana1* (FV3) | *Ranavirus* | *Ranavirus rana1* (FV3) | 0.0361 | Same species | - | - |
| Santee-Cooper ranavirus - HV_061_S43 | OQ718202.1 | *Alphairidovirinae* | *Ranavirus* | *Ranavirus micropterus1* (SCRAV) | *Ranavirus rana1* (FV3) | *Ranavirus* | *Ranavirus rana1* (FV3) | 0.0724 | Same species | - | - |
| Santee-Cooper ranavirus - HV2PWA_P1_S65 | OQ718203.1 | *Alphairidovirinae* | *Ranavirus* | *Ranavirus micropterus1* (SCRAV) | *Ranavirus rana1* (FV3) | *Ranavirus* | *Ranavirus rana1* (FV3) | 0.0690 | Same species | - | - |
| Santee-Cooper ranavirus - HV_TXLICA1_S64 | OQ718204.1 | *Alphairidovirinae* | *Ranavirus* | *Ranavirus micropterus1* (SCRAV) | *Ranavirus rana1* (FV3) | *Ranavirus* | *Ranavirus rana1* (FV3) | 0.0482 | Same species | - | - |
| Santee-Cooper ranavirus - JFB16_A072_S67 | OQ718205.1 | *Alphairidovirinae* | *Ranavirus* | *Ranavirus micropterus1* (SCRAV) | *Ranavirus rana1* (FV3) | *Ranavirus* | *Ranavirus rana1* (FV3) | 0.1814 | Same species | - | - |
| Santee-Cooper ranavirus - JR_FV3XL_S62 | OQ718206.1 | *Alphairidovirinae* | *Ranavirus* | *Ranavirus micropterus1* (SCRAV) | *Ranavirus rana1* (FV3) | *Ranavirus* | *Ranavirus rana1* (FV3) | 0.0219 | Same species | - | - |
| Santee-Cooper ranavirus - RM_LMRV_S86 | OQ718210.1 | *Alphairidovirinae* | *Ranavirus* | *Ranavirus micropterus1* (SCRAV) | *Ranavirus rana1* (FV3) | *Ranavirus* | *Ranavirus rana1* (FV3) | 0.0273 | Same species | - | - |
| Santee-Cooper ranavirus - RT117_hs | OQ718211.1 | *Alphairidovirinae* | *Ranavirus* | *Ranavirus micropterus1* (SCRAV) | *Ranavirus rana1* (FV3) | *Ranavirus* | *Ranavirus rana1* (FV3) | 0.0215 | Same species | - | - |
| Santee-Cooper ranavirus - RT126_hs | OQ718212.1 | *Alphairidovirinae* | *Ranavirus* | *Ranavirus micropterus1* (SCRAV) | *Ranavirus rana1* (FV3) | *Ranavirus* | *Ranavirus rana1* (FV3) | 0.0447 | Same species | - | - |
| Santee-Cooper ranavirus - RT128_hs | OQ718213.1 | *Alphairidovirinae* | *Ranavirus* | *Ranavirus micropterus1* (SCRAV) | *Ranavirus rana1* (FV3) | *Ranavirus* | *Ranavirus rana1* (FV3) | 0.0681 | Same species | - | - |
| Santee-Cooper ranavirus - RUK11_hs | OQ718214.1 | *Alphairidovirinae* | *Ranavirus* | *Ranavirus micropterus1* (SCRAV) | *Ranavirus rana1* (FV3) | *Ranavirus* | *Ranavirus rana1* (FV3) | 0.0415 | Same species | - | - |
| Santee-Cooper ranavirus - RUK13_hs | OQ718215.1 | *Alphairidovirinae* | *Ranavirus* | *Ranavirus micropterus1* (SCRAV) | *Ranavirus rana1* (FV3) | *Ranavirus* | *Ranavirus rana1* (FV3) | 0.0669 | Same species | - | - |
| Onychodactylus koreanus ranavirus - OKRV1 | PP518040.1 | *Alphairidovirinae* | *Ranavirus* | *Onychodactylus koreanus ranavirus* | *Ranavirus rana1* (FV3) | *Ranavirus* | *Ranavirus rana1* (FV3) | 0.0412 | Same species | - | [15] |
| Onychodactylus koreanus ranavirus - OKRV2 | PP518041.1 | *Alphairidovirinae* | *Ranavirus* | *Onychodactylus koreanus ranavirus* | *Ranavirus rana1* (FV3) | *Ranavirus* | *Ranavirus rana1* (FV3) | 0.0322 | Same species | - | [15] |
| Ranavirus sp. - CVASU FV3-like | OM287133.1 | *Alphairidovirinae* | *Ranavirus* | *Ranavirus* sp. | *Ranavirus rana1* (FV3) | *Ranavirus* | *Ranavirus rana1* (FV3) | 0.2008 | Same species | - | - |
| Mandarin fish ranavirus - NH-1609 | MG941005.3 | *Alphairidovirinae* | *Ranavirus* | *Mandarin fish ranavirus* | *Ranavirus micropterus1* (SCRAV) | *Ranavirus* | *Short-finned eel ranavirus* | 0.5871 | Same genus | - | [16] |
| Micropterus salmoides ranavirus - M2106 | OQ267587.1 | *Alphairidovirinae* | *Ranavirus* | *Micropterus salmoides ranavirus* | *Ranavirus micropterus1* (SCRAV) | *Ranavirus* | *Short-finned eel ranavirus* | 0.5826 | Same genus | - | [17] |
| Siniperca chuatsi ranavirus - 2207 | OQ267588.1 | *Alphairidovirinae* | *Ranavirus* | *Siniperca chuatsi ranavirus* | *Ranavirus micropterus1* (SCRAV) | *Ranavirus* | *Ranavirus perca1* (EHNV) | 0.5816 | Same genus | - | [17] |
| Siniperca chuatsi ranavirus | PV191281.1 | *Alphairidovirinae* | *Ranavirus* | *Siniperca chuatsi ranavirus* | *Ranavirus micropterus1* (SCRAV) | *Ranavirus* | *Short-finned eel ranavirus* | 0.5828 | Same genus | - | - |
| Mandarin fish ranavirus - MRV-ZQ17 | PV200168.1 | *Alphairidovirinae* | *Ranavirus* | *Mandarin fish ranavirus* | *Ranavirus micropterus1* (SCRAV) | *Ranavirus* | *Short-finned eel ranavirus* | 0.5814 | Same genus | - | [18] |
| Invertebrate iridovirus 25 - IIV-25 | HF920635.1 | *Betairidovirinae* | *Chloriridovirus* | *Invertebrate iridovirus 25* | *Chloriridovirus simulium2* (IIV25) | *Chloriridovirus* | *Chloriridovirus simulium2* (IIV25) | 0.0000 | Same species | - | [19) |
| Invertebrate iridescent virus - Kaz2018 | MT862761.1 | *Betairidovirinae* | *Iridovirus* | *Invertebrate iridescent virus Kaz2018* | *Iridovirus chilo1* (IIV6) | *Iridovirus* | *Iridovirus chilo1* (IIV6) | 0.2386 | Same genus | - | [20] |
| Iridovirus sp. - SocP20 | PP847201.1 | *Betairidovirinae* | *Iridovirus* | *Iridovirus* sp. | *Iridovirus chilo1* (IIV6) | *Iridovirus* | *Iridovirus chilo1* (IIV6) | 0.3869 | Same genus | - | [21] |

***** ISKNV: Infectious spleen and kidney necrosis virus; TSIV: Threespine stickleback iridovirus; SGIV: Singapore grouper iridovirus; FV3: Frog virus 3; CMTV: Common midwife toad virus; SCRAV: Santee-Cooper ranavirus; EHNV: Epizootic haematopoietic necrosis virus; ENARV: European North Atlantic ranavirus; IIV25: Invertebrate iridescent virus 25; IIV6: Invertebrate iridescent virus 6.

** The analysis based on the longest sequence (PQ335173.1) in the viral genome.

**References**

1. Yoxsimer AM, Offenberg EG, Katzer AW *et al*. Genomic sequence of the threespine stickleback iridovirus (TSIV) from wild *Gasterosteus aculeatus* in Stormy Lake, Alaska. *Viruses* 2024;16:1663. https://doi.org/10.3390/v16111663
2. Wang G, Xu W, Cheng H *et al*. Complete genome sequence and phylogenetic analysis of red seabream iridovirus isolated from a cage cultured small yellow croaker (*Larimichthys polyactis*) in China. *Arch Virol* 2022;167:2085–8. https://doi.org/10.1007/s00705-022-05508-5
3. Liu X, Chi H, Yang X *et al*. A comprehensive analysis of the genomic and proteomic profiles of a megalocytivirus isolated from *Larimichthys crocea*. *Front Microbiol* 2025;16:1528930. https://doi.org/10.3389/fmicb.2025.1528930
4. Koda SA, Subramaniam K, Francis-Floyd R *et al*. Phylogenomic characterization of two novel members of the genus *Megalocytivirus* from archived ornamental fish samples. *Dis Aquat Org* 2048;130:11–24. https://doi.org/10.3354/dao03250
5. Stöhr AC, López-Bueno A, Blahak S *et al*. Phylogeny and differentiation of reptilian and amphibian ranaviruses detected in Europe. *PLOS One* 2015;10:e0118633. https://doi.org/10.1371/journal.pone.0118633
6. Li W, Zhang X, Weng S *et al*. Virion-associated viral proteins of a Chinese giant salamander (*Andrias davidianus*) iridovirus (genus *Ranavirus*) and functional study of the major capsid protein (MCP). *Vet Microbiol* 2014;172:129–39. https://doi.org/10.1016/j.vetmic.2014.05.009
7. Claytor SC, Subramaniam K, Landrau-Giovannetti N *et al*. Ranavirus phylogenomics: signatures of recombination and inversions among bullfrog ranaculture isolates. *Virology* 2017;511:330–43. https://doi.org/10.1016/j.virol.2017.07.028
8. Borzym E, Stachnik M, Reichert M *et al*. Genome sequence of a ranavirus isolated from a red-eared slider (*Trachemys scripta elegans*) in Poland. *Microbiol Resour Ann* 2020;9:e00781–20. https://doi.org/10.1128/mra.00781-20
9. Liu F, Tian S, Feng Y *et al*. First report of a CMTV-like ranavirus in farmed *Percocypris pingi* in China. *Aquaculture* 2023;574:739701. https://doi.org/10.1016/j.aquaculture.2023.739701
10. Tian S, Yuan Y, Ouyang P *et al*. Isolation, identification and phylogenetic analysis of a ranavirus isolated from *Rana grylio*. *J Huazhong Agric Univ* 2025;44:258–64. https://doi.org/10.13300/j.cnki.hnlkxb.2025.02.026
11. Subramaniam K, Toffan A, Cappellozza E *et al*. Genomic sequence of a ranavirus isolated from short-finned eel (*Anguilla australis*). *Genome Ann* 2016;4:e00843–16. https://doi.org/10.1128/genomea.00843-16
12. Ariel E, Steckler NK, Subramaniam K *et al*. Genomic sequencing of ranaviruses isolated from turbot (*Scophthalmus maximus*) and Atlantic cod (*Gadus morhua*). *Genome Ann* 2016;4:e01393–16. https://doi.org/10.1128/genomea.01393-16
13. Conrad CRK, Subramaniam K, Chinchar VG *et al*. Genomic sequencing of ranavirus isolates from a three-spined stickleback (*Gasterosteus aculeatus*) and a red-legged frog (*Rana aurora*). *Microbiol Resour Ann* 2021;10:e00902–21. https://doi.org/10.1128/MRA.00902-21
14. Apakupakul K, Duncan M, Subramaniam K *et al*. Ranavirus (frog virus 3) infection in free-living three-toed box turtles (*Terrapene mexicana triunguis*) in Missouri, USA. *J Wildl Dis* 2024;60:151–63. https://doi.org/10.7589/JWD-D-23-00057
15. Kim J, Sung HW, Jung TS *et al*. First report of endemic frog virus 3 (FV3)-like ranaviruses in the Korean Clawed salamander (*Onychodactylus koreanus*) in Asia. *Viruses*, 2024;16:675. https://doi.org/10.3390/v16050675
16. Pan W, Liang M, You Y *et al*. Viral genomic methylation and the interspecies evolutionary relationships of ranavirus. *PLoS Pathog* 2024;20:e1012736. https://doi.org/10.1371/journal.ppat.1012736
17. Yu XD, Ke F, Zhang QY *et al*. Genome characteristics of two ranavirus isolates from mandarin fish and largemouth bass. *Pathogens* 2023;12:730. https://doi.org/10.3390/pathogens12050730
18. Zhang W, Duan C, Zhang H *et al*. Widespread outbreaks of the emerging mandarinfish ranavirus (MRV) both in natural and ISKNV-FKC vaccinated mandarinfish *Siniperca chuatsi* in Guangdong, South China, 2017. *Aquaculture* 2020;520:734989. https://doi.org/10.1016/j.aquaculture.2020.734989
19. Piégu B, Guizard S, Spears T *et al*. Complete genome sequence of invertebrate iridovirus IIV-25 isolated from a blackfly larva. *Arch Virol* 2014;159:1181–5. https://doi.org/10.1007/s00705-013-1918-x
20. Madina A, Andrey B, Elmira A *et al*. Complete genome sequence of invertebrate iridescent virus from Kazahstan. Research Square, https://doi.org/10.21203/rs.3.rs-1863387/v1, 25 July 2022, preprint: not peer reviewed.
21. Millerwise S, Lund MC, Schimidlin K *et al*. Coding complete genomes of an iridovirus and two parvoviruses identified in lab-reared social spiders (*Stegodyphus dumicola*). *Microbiol Resour Ann* 2024;13:e00739–24. https://doi.org/10.1128/mra.00739-24
